# Blood tissue Plasminogen Activator (tPA) of liver origin contributes to neurovascular coupling involving brain endothelial N-Methyl-D-Aspartate (NMDA) receptors

**DOI:** 10.1186/s12987-023-00411-w

**Published:** 2023-02-03

**Authors:** Jonathane Furon, Mervé Yetim, Elsa Pouettre, Sara Martinez de Lizarrondo, Eric Maubert, Yannick Hommet, Laurent Lebouvier, Ze Zheng, Carine Ali, Denis Vivien

**Affiliations:** 1 UNICAEN, INSERM UMR-S U1237, Physiopathology and Imaging of Neurological Disorders (PhIND), GIP Cyceron, Institut Blood and Brain @ Caen-Normandie (BB@C), Normandie University, Bvd Becquerel, BP 5229, 14074 Caen, France; 2 Department of Medicine, Medical College of Wisconsin, Milwaukee, WI USA; 3 Blood Research Institute, Versiti Blood Center of Wisconsin, Milwaukee, WI USA; 4 Department of Clinical Research, Caen-Normandie University Hospital, Caen, France

**Keywords:** NVC, tPA, NMDAR, GluN1, Liver

## Abstract

**Background:**

Regulation of cerebral blood flow (CBF) directly influence brain functions and dysfunctions and involves complex mechanisms, including neurovascular coupling (NVC). It was suggested that the serine protease tissue-type plasminogen activator (tPA) could control CNV induced by whisker stimulation in rodents, through its action on N-methyl-d-Aspartate receptors (NMDARs). However, the origin of tPA and the location and mechanism of its action on NMDARs in relation to CNV remained debated.

**Methods:**

Here, we answered these issues using tPA^Null^ mice, conditional deletions of either endothelial tPA (VECad-Cre^ΔtPA^) or endothelial GluN1 subunit of NMDARs (VECad-Cre^ΔGluN1^), parabioses between wild-type and tPA^Null^ mice, hydrodynamic transfection-induced deletion of liver tPA, hepatectomy and pharmacological approaches.

**Results:**

We thus demonstrate that physiological concentrations of vascular tPA, achieved by the bradykinin type 2 receptors-dependent production and release of tPA from liver endothelial cells, promote NVC, through a mechanism dependent on brain endothelial NMDARs.

**Conclusions:**

These data highlight a new mechanism of regulation of NVC involving both endothelial tPA and NMDARs.

**Supplementary Information:**

The online version contains supplementary material available at 10.1186/s12987-023-00411-w.

## Introduction

Functional hyperemia, also named neurovascular coupling (NVC), allows adequate local energy supply to brain cells, with key roles in the pathophysiology of the brain [1, 2]. For instance, brain disorders such as stroke or Alzheimer’s disease are associated with dysregulated NVC [3].

Initially described by its ability to activate plasminogen into plasmin in the blood [4], tissue-type Plasminogen Activator (tPA) is a serine protease widely expressed in the central nervous system (CNS) [5, 6], especially by neurons and endothelial cells [7, 8]. Extending its functions above the conversion of plasminogen into plasmin, tPA interferes with a variety of neuronal receptors [9], including N-Methyl-D-Aspartate receptors (NMDARs). Through these mechanisms, tPA is considered as a neuromodulator implicated in various brain functions and dysfunctions, including learning and memory processes [10, 11], anxiety behavior [12, 13] and neurovascular diseases such as stroke, in which it contributes to the homeostasis of the blood–brain barrier and to neuronal survival [14, 15].

Park et al. have proposed parenchymal tPA as an actor of NVC by its ability to increase NMDAR signaling [16] and proposed that the tPA/PAI-1 (type 1 plasminogen activator inhibitor) pathway could counteract the harmful neurovascular and cognitive effects of Aβ [17]. In parallel, Anfray et al. have proposed that circulating tPA could contribute to NVC [18]. Since in the CNS, tPA is expressed by endothelial cells and neurons and since both cell types also express NMDARs, the exact contribution of each actor to CNV modulation remains unclear [19–22]. Moreover, in addition to endothelial cells, hepatocytes are thought to be a major source of circulating tPA [23], and the levels of free tPA are counterbalanced by its inhibitor PAI-1 [24, 25]. Since altered levels of blood tPA and PAI-1 may contribute to several neurovascular diseases [17, 26, 27], they must be tightly controlled. There is a growing body of evidence that activation of endothelial luminal bradykinin receptors promotes the release of tPA by endothelial cells [28], whereas angiotensin-II leads to the release of PAI-1 [29]. Nevertheless, regulation of plasma levels of PAI-1 cannot be explained solely by the renin-angiotensin system [30].

These observations raise the possibility that modifications of the physiological levels of circulating tPA may impact NVC and subsequent brain functions/dysfunctions. Therefore, we further examined the role of tPA, especially circulating tPA, in functional hyperemia induced by whisker stimulation in mice, by using a large set of genetic tools, including tPA deficient mice, PAI-1 deficient mice, conditional deletion of either the endothelial tPA or for endothelial NMDARs and parabioses between wild type and tPA deficient mice. We thus demonstrate that vascular tPA, originating from liver endothelial cells, contributes to NVC through modulation of brain endothelial NMDAR signaling. Using parallel pharmacological and genetic approaches, we also demonstrate a bradykinin-dependent control of vascular tPA levels with direct impact on functional hyperemia.

## Materials and methods

### Animals

All experiments were conducted in accordance with the French ethical law (Decree 2013-118) and the European Communities Council guidelines (2010/63/EU). Protocols were approved by our local ethics committee dependent on the French Ministry of Research and Higher Education (agreement numbers Cenomexa #19208, #19978 and Ce5/2012/062). All applicable international, national, and/or institutional guidelines for the care and use of animals were followed.

All experiments were performed on 8–10 weeks old male C57BL/6 mice (Janvier Labs, Le Genest-Saint-Isle, France), tPA deficient (tPA^Null^) mice and their wild type (tPA^WT^) littermates (see “Mouse lines” section), VeCadCre/tPA^lox^ mice and their WT littermates (see “Mouse lines” section), VeCadCre/Grin1^lox^ mice and their WT littermates (see “Mouse lines” section) were bred in our animal facilities (CURB, Caen, France) and housed in a 12 h light/12 h dark cycle with free access to water and food.

### Mouse lines

Because of the large number of animals and mouse strains used in this study, we decided to use only males. We are nevertheless aware that collecting data from females would be important as well. All animals used were from the same genetic background (C57BL6J), and a corresponding littermate colony was used as a control for each genotype (homozygotic colonies maintained in parallel). tPA^Null^ mice and tPA^lox^ mice were generated by our group [31] in collaboration with the Mouse Clinical Institute (ICS, Illkirch, France, http://www.ics-mci.fr). To obtain tPA^Null^ mice, tPA^lox^ mice (C57BL6J background) in which exon 3 of the Plat gene was flanked by loxP sites were crossed with CMV-Cre mice to induce of Cre-mediated excision the third exon of the Plat gene in germline. PAI-1^−/−^ mice were obtained from professor H.R. Lijnen, Centre for Molecular and Vascular Biology, University of Leuven, Belgium. Grin1^lox^ mice (B6.129S4-Grin1tm2Stl/J; # 005246) were obtained from The Jackson Laboratory [32]. VE-Cadherin-Cre mice (B6.FVB-Tg(Cdh5-cre)7Mlia/J; # 006137) were obtained from F. Millat [33], Institute of Radioprotection and Nuclear Safety, Fontenay-aux-Roses, France. tPA^lox^ and Grin1^lox^ mice were crossed with VE-Cadherin-Cre mice to obtain VE-Cadherin-Cre/tPA^lox^ (VECad-Cre^ΔtPA^) and VE-Cadherin-Cre/Grin1^lox^ mice (VECad-Cre^ΔGluN1^).

### Pharmacological treatments

Treatments were injected intravenously. Recombinant tPA (rtPA) (10 mg/kg) prepared as previously described [18] was infused for 10 mins (a bolus of 150 µl for a total volume of 300 µl). rtPA was used at 10 mg/kg, instead of 0.9 mg/kg as used in humans, because human rtPA is ten-fold less active than murine tPA to activate mouse plasminogen. The selective bradykinin type 2 receptor agonist (B2Rag) ([Phe8 Ψ(CH-NH)-Arg9]-Bradykinin, Tocris) was used at 7, 15, 30 µg/kg, (0.1 ml for each dose) for CNV measurement or at 60 µg/kg (0.1 ml) for ELISA and 30 µg/kg (0.1 ml) for mean arterial pressure (MAP) measure. Angiotensin II (Angiotensin II acetate, Sigma, 1 µg/kg/min) was infused during 20 min to obtain a MAP of 120 mmHg for acute hypertension.

### Animal preparation prior to measurement of cerebral blood flow (CBF) variations induced by whisker stimulation

As previously described [18], animals were anesthetized using 5% isoflurane (Isoflurane Belamont) in 70% N_2_O/30% O_2_. Mice were intubated and placed under mechanical ventilation (120 BPM, 10 ml/kg) by maintaining anesthesia with 2% isoflurane in 70% N_2_O/30% O_2_. The caudal vein and femoral artery were catheterized for pharmacological injections (see “Pharmacological treatments” section) and to evaluate physiological parameters: pCO_2_, pH and MAP. Mice were then placed in a stereotaxic frame. An incision was made along the midline head skin to expose the skull and lidocaine (Xylocaine, 5% spray^®^, AstraZeneca) was sprayed on the head. Whiskers on the left side were cut to let a length of 1 cm. Anesthesia was switched with subcutaneous infusion of medetomidine (Domitor^®^, Pfizer, 0.1 mg/kg) then isoflurane, N_2_O and O_2_ were stopped ten minutes later. CBF were recorded after only 20 min to allow isoflurane’s effects dissipation and CBF stabilization. Physiological parameters were measured in parallel experiments (Additional file 1: Fig. S1). We did not use ketamine, has it has effects on NMDARs.

### Laser Doppler Speckle Flowmetry

Laser Doppler Flowmetry (LDF) was used to measure relative CBF during whisker stimulations, of the whole surface of the brain with intact skull. Acquisitions were made with a laser speckle contrast imager (MoorFLPI-2, Moor Instrument, Exposure Time: 20 s, Filter: 25 frames, Sample Interval: 1000 ms, Image Resolution: 752 × 580). The whole left whiskers were mechanically shaken (4 Hz) during 30 s for three times separated by 60 s of resting. Analysis was made with MoorFLPI-2 Review V5.0 software, stimulation frames and resting frames were averaged and subtracted to obtain an activation map corresponding to the barrel cortex. CBF in this area was calculated to obtain a percentage of CBF change from baseline, then the three stimulations were averaged together.

### Parabiosis

The protocol has been improved from a former study [34]. Mice were anesthetized (see animal preparation section), and received buprenorphine (Buprecare^®^, Axience, 0.1 mg/kg) before proceeding with the surgery. Future parabionts were placed in the lateral position, back-to-back. A longitudinal skin incision was performed, the skin was gently detached, and joints were attached with non-absorbable sutures. The skins of the two mice were connected with non-absorbable sutures. Controls were performed to assess the efficacy of parabiosis, using measurements of blood glucose levels and DOTA-Gd MRI analyses (Additional file 1: Fig. S2).

### Partial hepatectomy

Animals were anesthetized (see animal preparation section), and received buprenorphine (Buprecare^®^, Axience, 0.1 mg/kg). Briefly, a transverse laparotomy was made. The left lateral lobe, left and right median lobes were ligatured using non-resorbable sutures, allowing a resection of around 70% of the total liver. For sham conditions, only the transverse laparotomy was made. Experiments were performed 24 h after partial hepatectomy (see “Laser Doppler Speckle” section), which did not influence mean arterial blood pressure (Additional file 1: Fig. S4).

### ELISA for tPA

Plasma total tPA levels from mice were analysed using commercial ELISA kits (Innovative Research, Inc.), according to the manufacturer’s instructions. Blood samples were collected by intracardiac puncture as previously described [18].

### Immunohistochemistry

Deeply anesthetized mice were transcardially perfused with cold heparinized saline (15 ml/min), then with 4% paraformaldehyde in 0.1 M sodium phosphate buffer, pH 7.4 (150 ml, 15 ml/min). Brain and liver sections were incubated overnight at room temperature with primary antibodies rabbit anti-tPA (1:1500, generous gift from R. Lijnen, Leuven) and rat anti-CD31 (1:1000, 553370, BD Biosciences). Primary antibodies were revelated using Fab’2 fragment anti-rabbit IgG linked to CY3, and anti-rat IgG linked to FITC (1:800, Jackson ImmunoReasearch) co-incubated 90 min at room temperature, then coverslipped using mounting medium containing DAPI. Images were digitally captured using an epifluorescence microscope (Leica DM6000). Images were assessed using ImageJ software (NIH).

### In situ hybridization

#### Tissue preparation

All surfaces and tools were cleaned with RNAsezap^®^ (Sigma) to ensure that the tissues were not contaminated with RNases. The mice were deeply anesthetized and intracardiacally perfused with a cold saline solution supplemented with 0.1% heparin. After exsanguination, brains and livers were dissected and directly embedded in the tissue embedding agent tek-OCT (Sakura) and frozen in isopentane cooled to − 70 °C using dry ice. The tissues were then cut with a cryostat (CM3050, Leica Microsystems) and 10 µm sections were conserved at − 80 °C until use.

#### In situ hybridization

The RNAscope TM Multiplex Fluorescent V2 kit (Biotechne, MN, USA) was used according to the manufacturer's instructions. All laboratory solutions were prepared in advance with autoclaved water at 0.1% DEPC (Sigma-Aldrich, MO, USA; D5758) to avoid any contamination by RNAses and DNAses. Our target was revealed with the *Plat* probe, revealing the *plat* gene encoding tPA (Biotechne, MN, USA; 586951) coupled with Opal 520 fluorophore (Akoya, MA, USA; SKU FP1487001KT; 1/5000). At the end of the protocol, we carried out immunostaining steps with the primary antibodies incubated overnight at 4 °C (anti-CD31 antibody, BD Biosciences; 555024 1/500; anti-phalloidin antibody, Invitrogen N21479 1/300). After 3 washes in 1 × PBS, the appropriate secondary antibodies coupled with Cyanine 3 or Cyanine 5 (1/800 Jackson Immunoresearch, West Grove, CA, USA) were incubated at room temperature for 90 min. Once washed, the slides were mounted with coverslip and mounting medium with DAPI (Fluoromont-G; Thermofisher, MA USA; 15596276).

### Statistical analyses

Results are expressed as box plots with medians, 1st and 3rd quartiles, min and max with values for each mouse. Time courses represent the mean ± the standard error of the mean (SEM) in transparency. Statistical analysis was performed using Mann–Whitney test, Kruskal Wallis test or ANOVA with Tukey test with GraphPad software. Data were considered statistically different if probability values (p) were at least < 0.05.

## Results

### The tPA/PAI-1 axis influences functional hyperemia induced by whisker stimulation

**Fig. 1:**
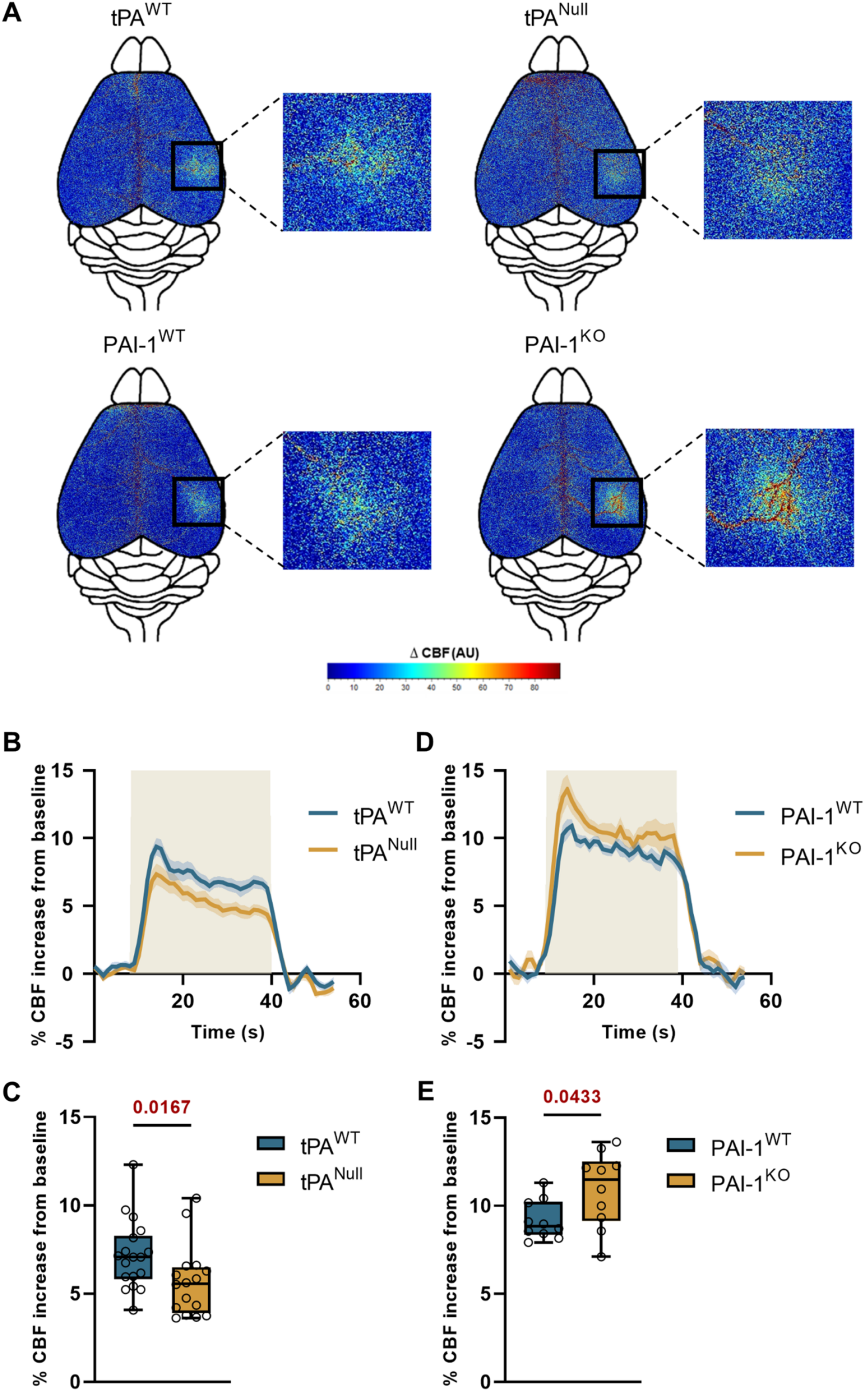
The tPA/PAI-1 axis controls NVC. **A** Colormap corresponds to the activation map related ∆CBF changes during whisker stimulations of tPA^Null^, PAI-1^KO^ mice and their wild type littermates. Maps were obtained by subtraction of stimulation frames and resting frames. Warm colours indicate an elevation of CBF during whisker stimulations. **B**, **D** Time course of % CBF increase (mean ± SEM) during whisker stimulations () of tPA^Null^ (**B**) and PAI-1^−/−^ (**D**) mice () and their littermates (). **C**, **E** Box plots show the variations of % CBF increase from baseline during whisker stimulations of tPA^Null^ (**C**) and PAI-1^−/−^ (**E**) mice and their littermates. Box plots with medians, 1st and 3rd quartiles, min and max with values for each mouse. *p < 0.05 from tPA^WT^ or PAI-1^WT^, Mann–Whitney test, n = 18/16 tPA^WT^/tPA^Null^, n = 10 PAI-1^WT^/PAI-1^KO^

We performed our experiments by maintaining the skull intact, to avoid any artefact due to craniectomy. Also important, animals were maintained sedated using medetomidine instead of ketamine which interferes with NMDAR signaling. After confirming by immunostainings (IHC) and in situ hybridization (ISH) the constitutive deletion of tPA in Null mice (Additional file 1: Figs. S9, S10), we showed that the CBF increase evoked by whisker stimulation was significantly impaired in tPA^Null^ mice when compared to their WT littermates (Fig. 1B, C) (+ 5.65 ± 0.49% for tPA^Null^ mice, n = 16, vs. + 7.16 ± 0.46% of CBF increase for WT mice, n = 18, i.e. 21% lower CBF increase for tPA^Null^ mice compared to tPA^WT^ mice, p-value 0.0167; each dot corresponds to the mean of 3 stimulations per animal). Conversely, the CBF increase evoked by whisker stimulation was significantly bigger in PAI-1-deficient mice (PAI-1^KO^) when compared to their WT littermates (Fig. 1D, E) (+ 10.9 ± 0.67% for PAI-1^KO^ mice, n = 10, vs. + 9.17 ± 0.35% of CBF increase for PAI-1^WT^ mice, n = 10, i.e. + 19.1% of CBF increase for PAI-1^KO^ mice compared to PAI-1^WT^ mice, p-value 0.0433). No modification of the baseline was observed between groups (this was also true for the other experiments presented in this study). These data demonstrate that the endogenous ratio tPA/PAI-1 tunes the efficiency of NVC.

### Circulating tPA physiologically influences NVC

**Fig. 2:**
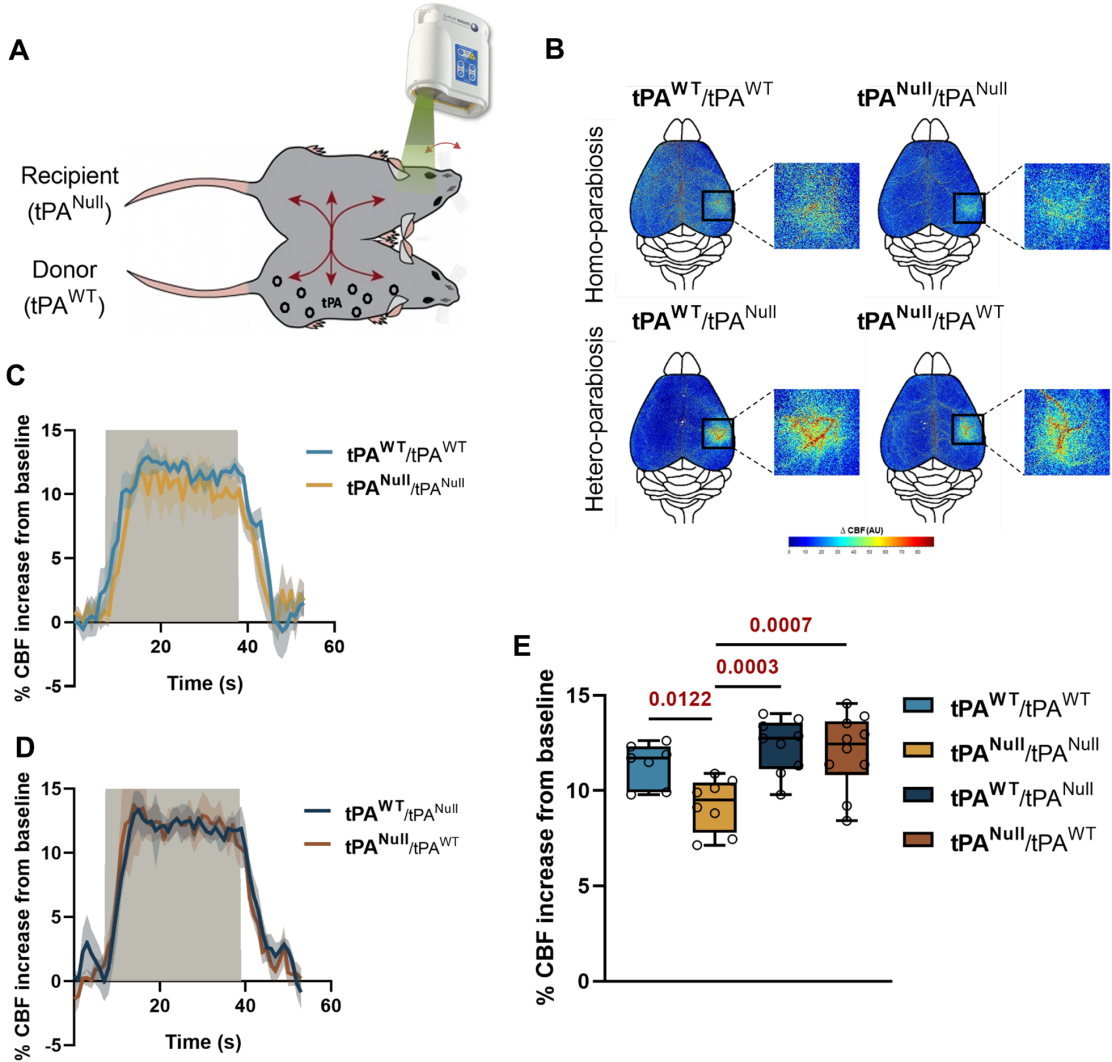
Endogenous tPA levels influence NVC. **A** Schematic representation of the procedure of whisker stimulations paradigm between two mice (tPA^Null^ and tPA^WT^) in parabiosis. tPA from the donor (tPA^WT^) will be share to the recipient (tPA^Null^). **B** Colormap corresponds to the activation map related ∆CBF changes during whisker stimulations in parabiont tPA^Null^ or tPA^WT^ mice. Warm colours indicate an elevation of CBF during whisker stimulations. **C**, **D** Time course of % CBF increase (mean ± SEM) during whisker stimulations () in homo-parabiosis tPA^WT^ () and tPA^Null^ () mice (**C**) and hetero-parabiosis tPA^WT^ () and tPA^Null^ () mice (**D**). **E** Box plots show the variation of % CBF increase from baseline during whisker stimulations in homo-parabiosis **tPA**^**WT**^/tPA^WT^ and **tPA**^**Null**^/tPA^Null^, and hetero-parabiosis **tPA**^**WT**^/tPA^Null^ and **tPA**^**Null**^/tPA^WT^ (the stimulated mouse is mentioned in bold). Box plots with medians, 1st and 3rd quartiles, min and max with values for each mouse. *p < 0.05 and from **tPA**^**Null**^/tPA^Null^, one-way ANOVA and Uncorrected Fisher’s LSD tests, n = 7 **tPA**^**WT**^/tPA^WT^, n = 8 **tPA**^**Null**^/tPA^Null^, n = 9 **tPA**^**WT**^/tPA^Null^, n = 10 **tPA**^**Null**^/tPA^WT^ (n represents the number of stimulated animals)

To ascertain that normal levels of circulating tPA control CNV, we used a model of parabiosis between tPA^WT^ and/or tPA^Null^ mice (Fig. 2A, and Additional file 1: Fig. S2). tPA^WT^/tPA^WT^, tPA^Null^/tPA^Null^ and tPA^WT^/tPA^Null^ parabiontic couples were maintained during a period of 3 weeks, prior to whisker stimulation-induced NVC assays (the stimulated animal appears in bold). As reported for individual animals, tPA^Null^ mice (**tPA**^**Null**^) from homotypic pairs (**tPA**^**Null**^/tPA^Null^), had an impaired NVC compared to **tPA**^**WT**^ mice from homotypic pairs (**tPA**^**WT**^/tPA^WT^) (Fig. 2B, D) (+ 9.2 ± 0.4% for **tPA**^**Null**^/tPA^Null^ mice, n = 8, vs. + 11.3 ± 0.4% of CBF increase for **tPA**^**WT**^/tPA^WT^ mice, n = 7, i.e. -18.5% of CBF increase for **tPA**^**Null**^/tPA^Null^ mice compared to **tPA**^**WT**^/tPA^WT^ mice, p-value 0.0122). Interestingly, parallel experiments performed on tPA^Null^ mice of hetero-parabiosis (tPA^Null^/tPA^WT^) animals, revealed a rescue of NVC to levels similar to the tPA^WT^ animals (Fig. 2B–E) (+ 12 ± 0.6% of CBF increase for tPA^Null^ mice from a pair of **tPA**^**Null**^/tPA^WT^ mice, n = 10, vs. + 9.2 ± 0.4% for tPA^Null^ mice from a pair of **tPA**^**Null**^/tPA^Null^ mice, n = 8, p-value 0.0007). All together, these data demonstrate that endogenous levels of circulating tPA directly influence NVC.

**Fig. 3:**
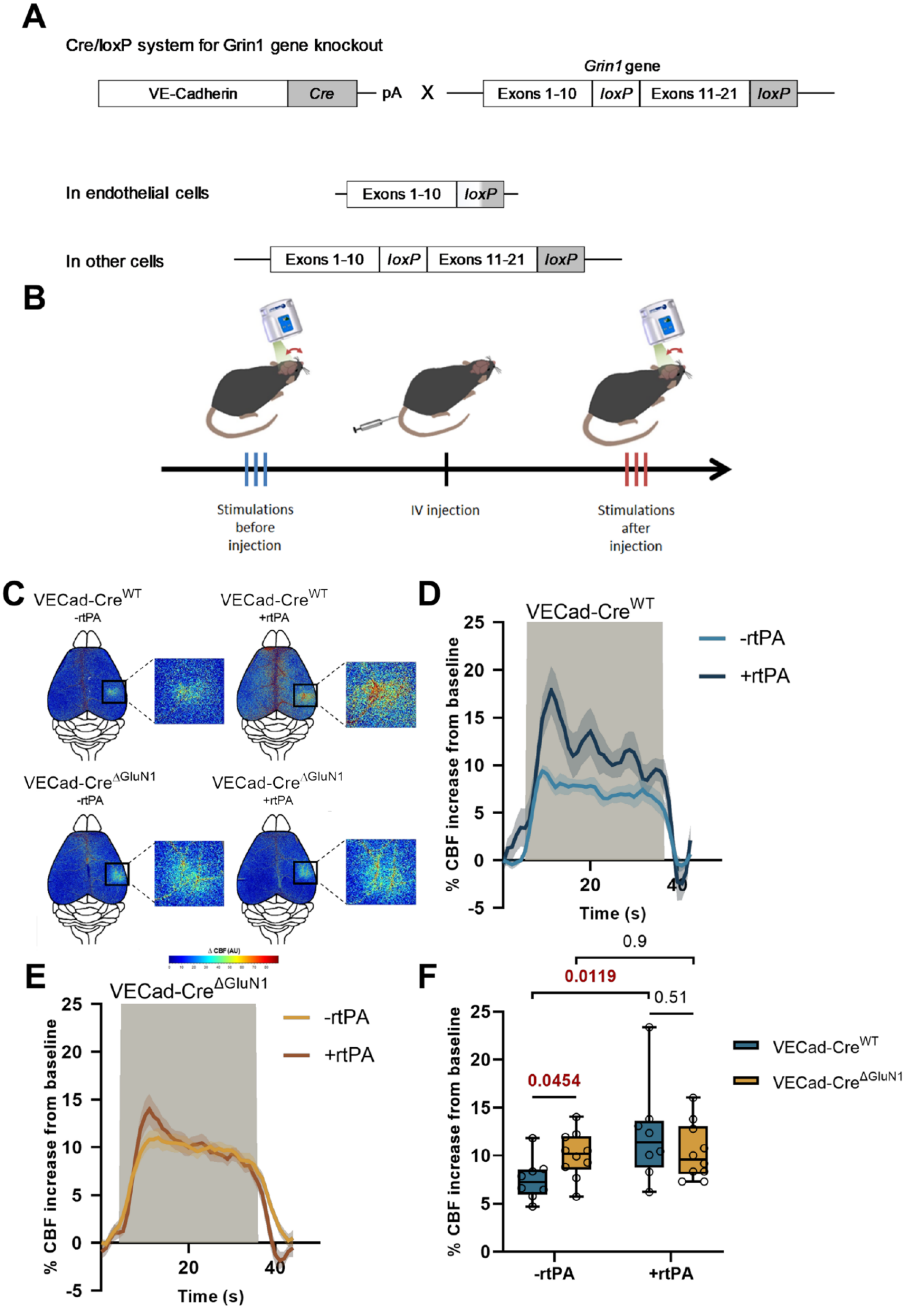
Endothelial NMDAR controls NVC, an effect mediated by vascular tPA. **A** Schematic representation of the generation of VE-Cadherin Cre/Grin-1 Lox mice. The cadherin 5 promoter was used to drive expression of CRE recombinase in the vascular endothelium. LoxP sites were flanked in the transmembrane and C-term regions of Grin-1 gene (exons 11–21). This configuration carries out the deletion of endothelial Grin-1 gene while conserving Grin-1 in other cells. **B** Schematic representation of the experimental timeline of whisker stimulation paradigm: 3 trains of stimulations were made on laser speckle flowmetry before IV infusion of rtPA for 10 min and 3 additional trains after (control group corresponds to an IV infusion of HEPES buffer). **C** Colormap corresponds to the activation map related ∆CBF changes during whisker stimulations in VECad-Cre^ΔGluN1^ mice and their littermate before and after IV infusion of rtPA. Warm colours indicate an elevation of CBF during whisker stimulations. **D**, **E** Time course of % CBF increase (mean ± SEM) during whisker stimulations () in VECad-Cre^ΔGluN1^ mice (**E**) and their littermate (**D**), before (/) and after (/) IV infusion of rtPA. **F** Box plots show the variations of % CBF increase from baseline during whisker stimulations in VECad-Cre^ΔGluN1^ mice and their wild type littermates before and after IV infusions of rtPA. Box plots with medians, 1st and 3rd quartiles, min and max with values for each mouse. *p < 0.05 from VECad-Cre^WT^ or -rtPA, Kruskal–Wallis and Uncorrected Dunn’s tests, n = 8 VECad-Cre^WT^, n = 10 VECad-Cre^ΔGluN1^

### Endothelial NMDA receptors mediate the vascular tPA-dependent NVC induced by whisker stimulation

Our data first reveal an increased neurovascular coupling in VECad-Cre^ΔGluN1^ mice [20, 32] (conditional deletion of endothelial GluN1, Fig. 3A, see Additional file 1: Fig. S11 for control ISH which confirms the conditional deletion of GluN1 in endothelial cells) compared to VECad-Cre^WT^ control mice (Fig. 3C–F) (+ 10 ± 0.7% for VECad-Cre^ΔGluN1^ mice, n = 10, vs. + 7.5 ± 0.8% of CBF increase for VECad-Cre^WT^ mice, n = 8, i.e. + 33.8% of CBF increase for VECad-Cre^ΔGluN1^ mice compared to VECad-Cre^WT^ mice, p-value 0.0454). Although the intravenous injection of rtPA (10 mg/kg) led to an increased NVC in wild type (VECad-Cre^WT^) mice, it did not in VECad-Cre^ΔGluN1^ mice (Fig. 3C–F) (+ 12.2 ± 1.8% of CBF increase for VECad-Cre^WT^ mice treated by rtPA, n = 8, vs. + 7.4 ± 0.8% for untreated VECad-Cre^WT^ mice, n = 10, i.e. + 62.2% of CBF increase VECad-Cre^WT^ mice treated with rtPA compared to untreated VECad-Cre^WT^ mice, p-value 0.0119, + 3% of CBF increase for VECad-Cre^ΔGluN1^ mice treated with rtPA compared to untreated VECad-Cre^ΔGluN1^ mice, p-value > 0.999). This set of original data demonstrates first that circulating tPA drives NVC-induced by whisker stimulation in mice; and second, that endothelial NMDARs are required to mediate this effect of vascular tPA.

### Activation of the bradykinin pathway promotes release of tPA in the blood stream and neurovascular coupling

**Fig. 4:**
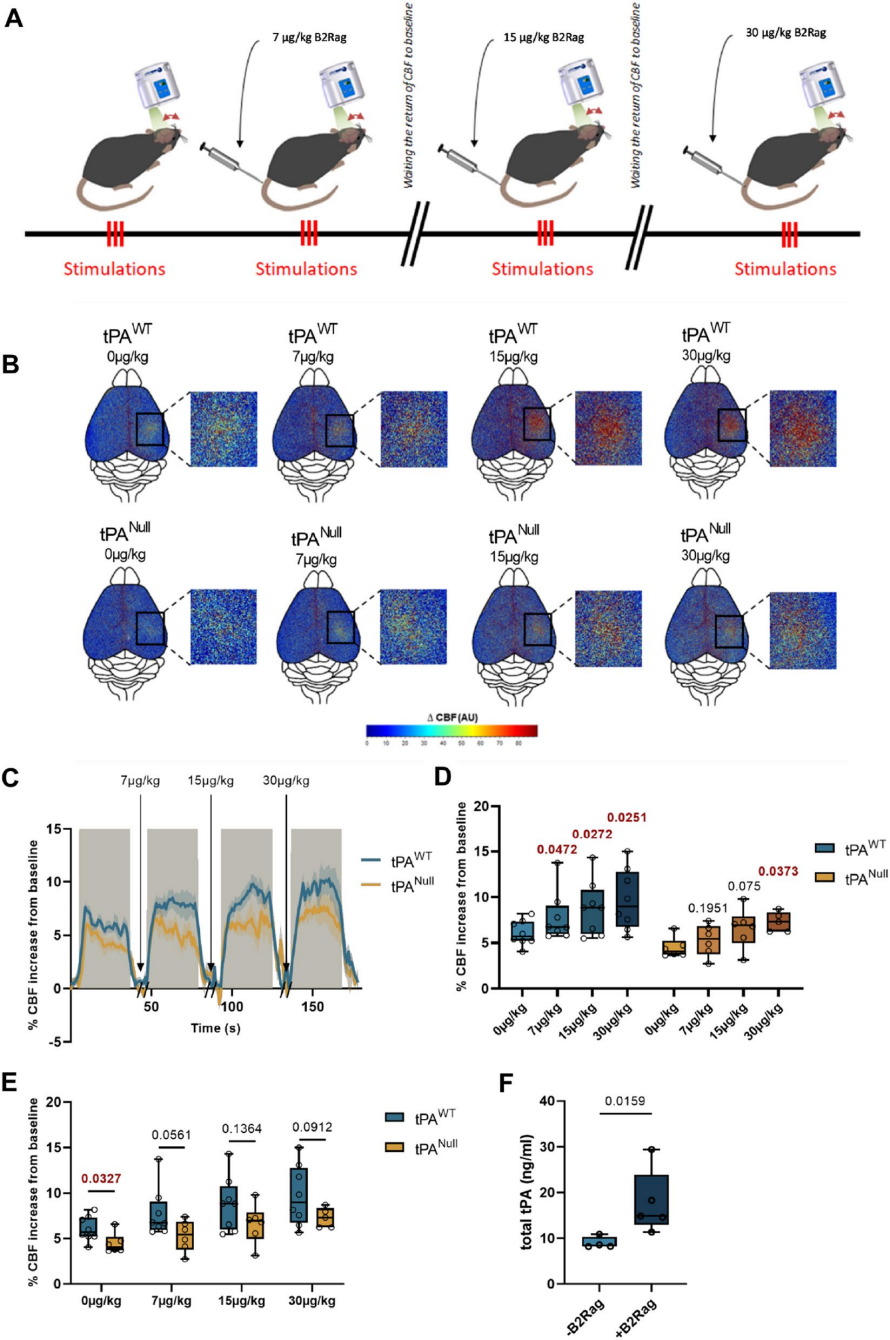
Intravenous administration of bradykinin type 2 receptor agonist (B2Rag) influences NVC in a tPA-dependent manner. **A** Schematic representation of the experimental timeline of whisker stimulation paradigm: 3 trains of stimulations were made on laser speckle flowmetry 10 min after IV injection of B2Rag at different doses (0, 7, 15 and 30 µg/kg). Between each dose, the return of CBF to baseline was waited. **B** Colormap corresponds to the activation map related ∆CBF changes during whisker stimulation in tPA^Null^ mice and their wild type littermates for the different doses of B2Rag (0, 7, 15 and 30 µg/kg). Warm colours indicate an elevation of CBF during whisker stimulations. **C** Time course of % CBF increase (mean ± SEM) during whisker stimulations () in tPA^Null^ mice () and their littermate (), at different doses of B2Rag (0, 7, 15 and 30 µg/kg). **D**, **E** Box plots show the variations of % CBF increase from baseline during whisker stimulation in tPA^Null^ mice and their wild type littermates for the different doses of B2Rag (0, 7, 15 and 30 µg/kg). Box plots with medians, 1st and 3rd quartiles, min and max with values for each mouse. *p < 0.05 from 0 mg/kg or tPA^WT^, Two-Way-ANOVA and Uncorrected Fisher’s LSD tests, n = 8 tPA^WT^, n = 6 tPA^Null^. **F** Box plots representing ELISA analyses of total tPA in tPA^WT^ mice treated or not with the B2Rag. A saline solution or 60 µg/kg of B2Rag were injected intravenously and plasma samples were collected 5 min later and subjected to ELISA analysis. Box plots with medians, 1st and 3rd quartiles, min and max with values for each mouse. *p < 0.05 from Vehicle, Mann–Whitney test, n = 4 Vehicle, n = 5 B2Rag

In order to further investigate how endothelial function/dysfunction may contribute to the ability of vascular tPA to modulate NVC, we decided to test how the angiotensin/bradykinin couple (and related pathways) may be involved, respectively reported to contribute to the regulation of the levels of PAI-1 and tPA in the circulation [35, 36]. Thus, animals subjected to whisker stimulation-induced NVC were previously subjected to intravenous injections of either [Phe8Ψ(CH-NH)-Arg9]-Bradykinin (see methods section) as an agonist for bradykinin type II receptors (B2Rag) (Fig. 4A) or angiotensin-II (Ang-II, 1 µg/kg/min) (see methods section, Additional file 1: Fig. S6A). As expected, the B2R agonist (30 µg/kg) led to a decrease in basal mean arterial blood pressure (60 ± 4 mmHg for B2Rag treated animals vs. 91.5 ± 2.9 mmHg for control animals, n = 4 per group, p-value 0.0286, Additional file 1: Fig. S3) and angiotensin-II (1 µg/kg/min) led to an increase in basal arterial blood pressure (122.5 ± 4.7 mmHg for Angiotensin-II treated animals vs. 85.75 ± 4 mmHg for control animals, n = 4 per group, p-value 0.0286, without affecting physiological parameters (Additional file 1: Fig. S5). Using this procedure, we showed that the CBF increase evoked by whisker stimulation was not significantly modified in angiotensin-II treated wild type animals (Additional file 1: Fig. S6B–E) (+ 9 ± 0.6% of CBF increase for tPA^WT^ mice treated with Ang-II, n = 10, vs. + 10.1 ± 0.3% of CBF increase for tPA^WT^ mice, n = 10, p-value 0.1067), but that it was significantly increased in B2Rag-treated animals compared to the control non treated group (Fig. 4 B–E) (+ 6 ± 0.4% of CBF increase for tPA^WT^ mice, n = 8, vs. + 9.7 ± 1.1% of CBF increase for tPA^WT^ treated with B2Rag at 30 µg/kg, n = 8, p-value 0.0251). We then compared tPA^WT^ and tPA^Null^ mice, treated intravenously with increasing concentrations of B2Rag (0, 7, 15, 30 µg/kg). Our data (Fig. 4B–E) clearly revealed a dose-dependent potentiation of NVC by the agonist of B2R in tPA^WT^ animals and no effect in tPA^Null^ mice (+ 18.4% of CBF increase for tPA^Null^ mice at 7 µg/kg of B2Rag, n = 6, p-value 0.1951, vs. + 28.5% of CBF increase for tPA^WT^ mice, n = 8 at 7 µg/kg of B2Rag, p-value 0.0472, when compared to the non-treated tPA^Null^ or tPA^WT^ mice). Parallel experiments were performed to estimate the levels of circulating tPA in the circulation of tPA^WT^ mice subjected to 60 µg/kg of B2Rag (Fig. 4F). In agreement with our previous data on NVC, B2Rag led to an increase in vascular tPA (+ 8.7 ng/ml of total tPA after injection of B2Rag, n = 4–5, p-value 0.0159, Fig. 4F). Altogether, these data reveal that activation of the bradykinin pathway leads to an increased release of tPA in the blood stream and subsequent potentiation of whisker stimulation-induced NVC.

### tPA released from liver endothelial cells contributes to the regulation of NVC

**Fig. 5:**
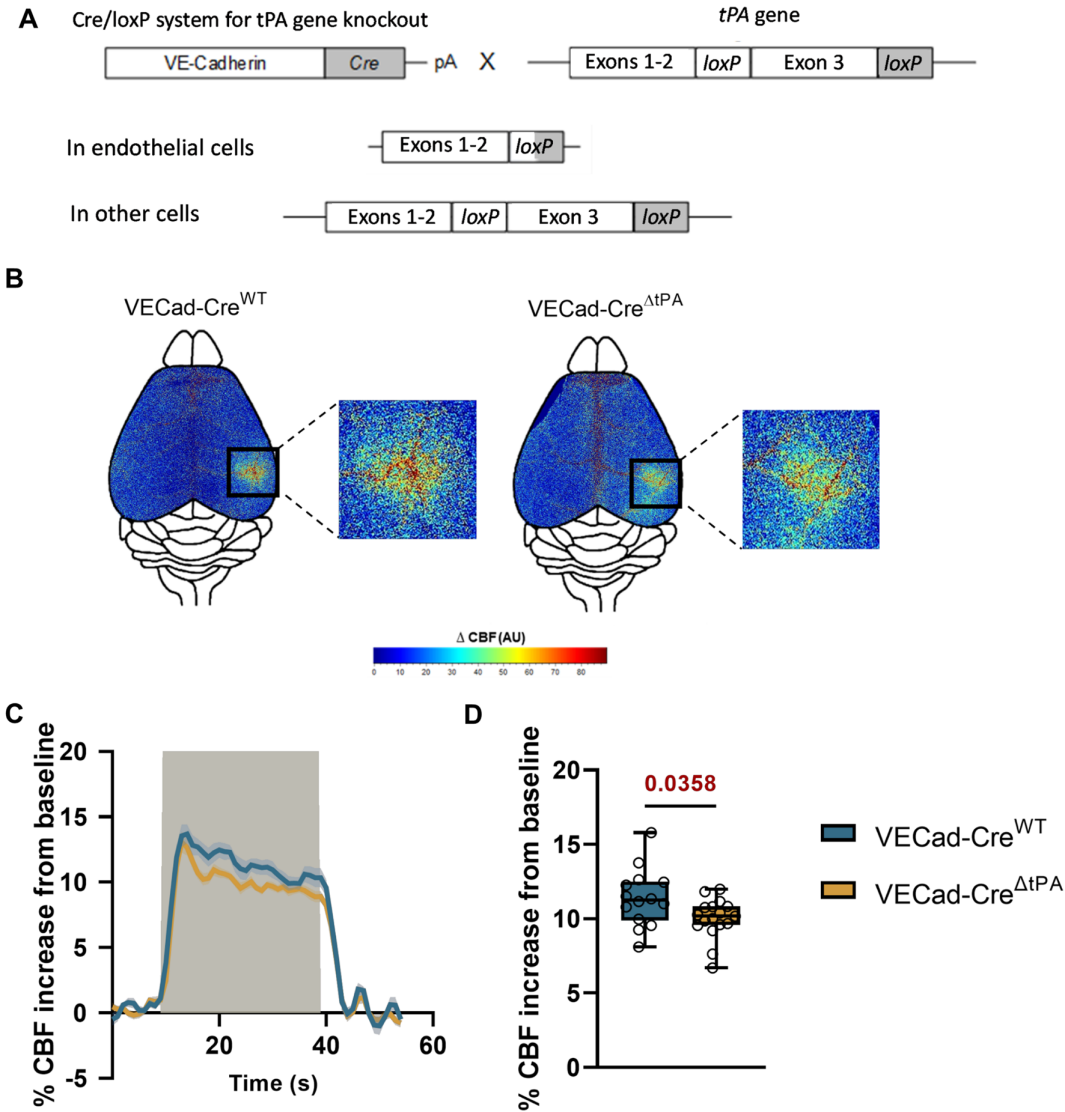
Endothelial tPA controls NVC. **A** Schematic representation of the generation of VE-Cadherin Cre/tPA Lox mice. The cadherin 5 promoter was used to drive expression of Cre recombinase in the vascular endothelium. LoxP sites were flanked in the exon 3 of tPA gene. This configuration carries out the deletion of endothelial tPA gene while conserving tPA in other cells. **B** Colormap corresponds to the activation map related ∆CBF changes during whisker stimulations of VECad-Cre^ΔtPA^ mice and their wild type littermates. Maps were obtained by subtraction of stimulation frames and resting frames. Warm colours indicate an elevation of CBF during whisker stimulations. **C** Time course of % CBF increase (mean ± SEM) during whisker stimulations () of VECad-Cre^ΔtPA^ () and their littermates (). **D** Box plots show the variations of % CBF increase from baseline during whisker stimulation of VECad-Cre^ΔtPA^ mice and their littermates. Box plots with medians, 1st and 3rd quartiles, min and max with values for each mouse. *p < 0.05 from VECad-Cre^WT^, Mann–Whitney test, n = 14/17 VECad-Cre^WT^/ VECad-Cre^ΔtPA^

**Fig. 6:**
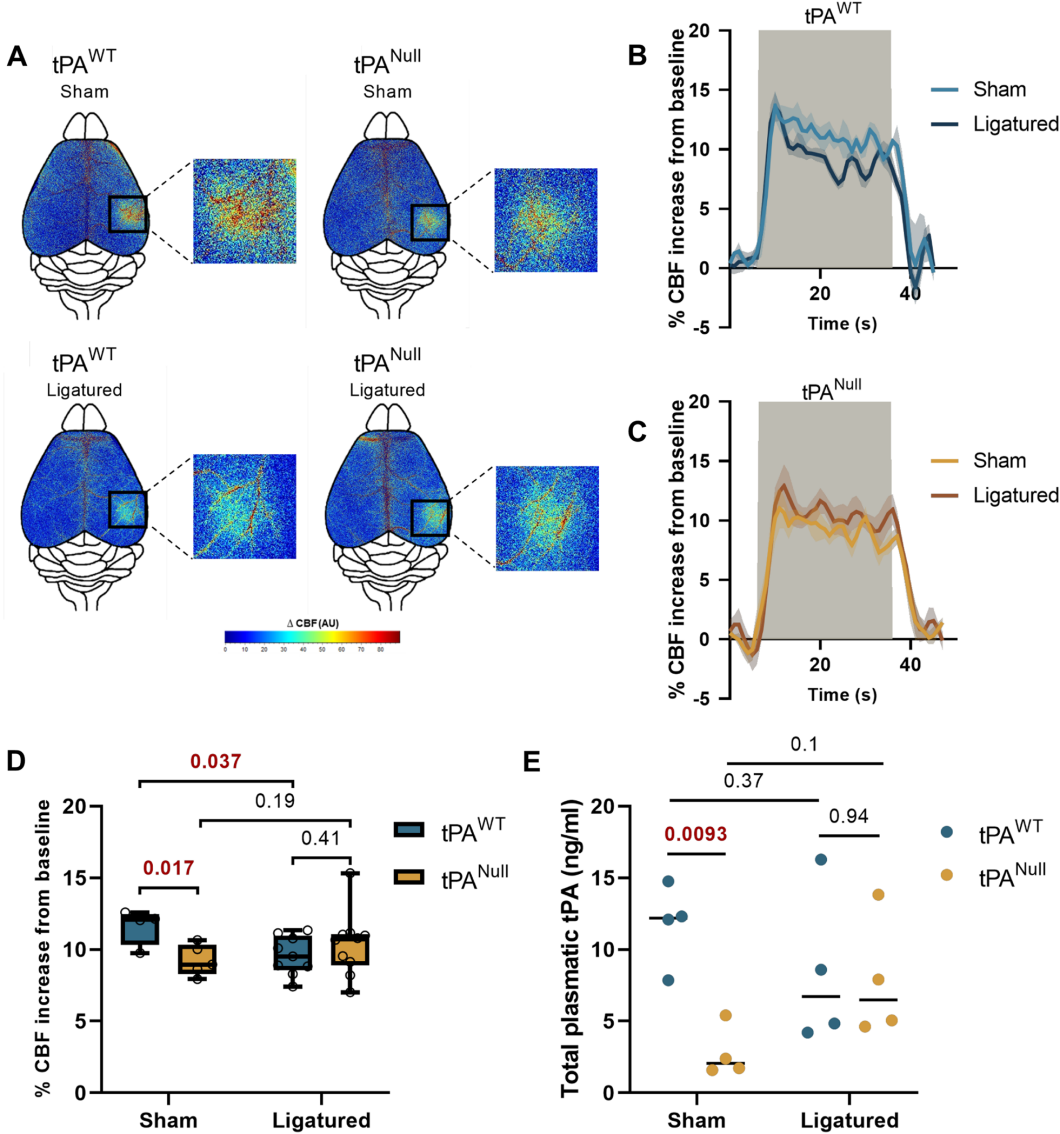
Partial hepatectomy influences NVC. **A** Colormap corresponds to the activation map related ∆CBF changes during whisker stimulations in tPA^Null^ mice and their littermate in sham and ligatured conditions 24 h after partial hepatectomy. Warm colours indicate an elevation of CBF during whisker stimulations. **B**, **C** Time course of % CBF increase (mean ± SEM) during whisker stimulations () in tPA^Null^ mice (**C**) and their littermate (**B**), in sham (/) or ligatured (/) conditions. **D** Box plots show the variation of % CBF increase from baseline during whisker stimulations in tPA^Null^ mice and their littermate in sham and ligatured conditions. Box plots with medians, 1st and 3rd quartiles, min and max with values for each mouse. **E** Mean ± SEM representing ELISA analyses of total tPA in tPA^Null^ mice and their littermate in sham and ligatured conditions (n = 4 for each group). 24 h after partial hepatectomy, plasma samples were collected and subjected to ELISA analysis. *p < 0.05 from sham tPA^WT^, Kruskal–Wallis and Uncorrected Dunn’s tests, n = 4 sham tPA^WT^, n = 9 ligatured tPA^WT^, n = 5 sham tPA^Null^, n = 10 ligatured tPA^Null^

**Fig. 7:**
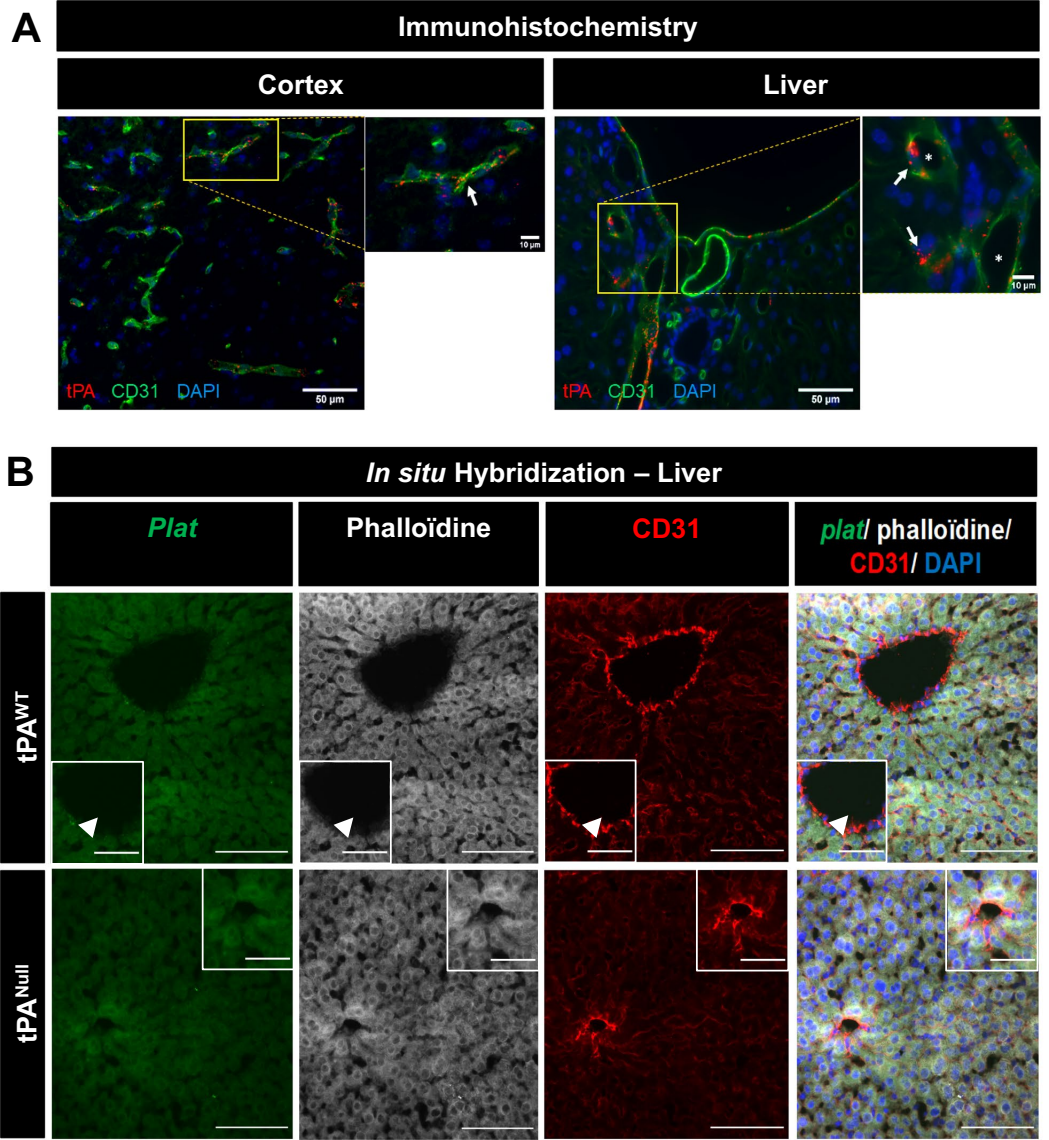
tPA is present in brain and liver endothelial cells. **A** Epifluorescence images of brain cortex and liver slices from C57BL/6 adult mice (Scale bar = 50 µm, zooms = 10 µm) revealing tPA (red), CD31 (green) and cells nuclei (DAPI, blue). tPA was observed in endothelial cells (arrow) in both the brain cortex and liver. Lumen of vessels was represented by stars. **B** Hepatocytes do not express tPA, but liver endothelial cells do. Representative photomicrographs of liver tissue sections subjected to in situ hybridization to reveal plat mRNA (mRNA encoding for the gene tPA; green), phalloïdine (hepatocyte marker; grey) and CD31 (red) staining; scale bar 100 µm and 50 µm in the magnified inserts. Images were digitally captured using a camera (CoolSNAP; Photometrics) and/or with an inverted confocal microscope (SP5, Leica). Images were visualized respectively with Metavue 5.0 software (Molecular Devices, USA) and LAS AF lite software (LEICA)

We then investigated the cellular origin of the circulating tPA involved in the control of NVC. Our data reveal a reduced NVC in VECad-Cre^ΔtPA^ mice, having a conditional deletion of endothelial tPA [33] (Fig. 5A, see Additional file 1: Fig. S12 for control ISH which confirms the conditional deletion of tPA in endothelial cells), compared to VECad-Cre^WT^ control mice (+ 10.02 ± 0.3% for VECad-Cre^ΔtPA^ mice, n = 17, vs. + 11.4 ± 0.5% of CBF increase for VECad-Cre^WT^ mice, n = 14, i.e.  − 12% of CBF increase for VECad-Cre^ΔtPA^ mice compared to VECad-Cre^WT^ mice, p-value 0.0358, Fig. 5B–D). This set of data demonstrate that tPA produced and released by endothelial cells directly contributes to the modulation of NVC. Since tPA is also expressed from liver, possibly from hepatocytes [37], we investigated whether partial hepatectomy (see methods section) may influence NVC induced by whisker stimulation (Fig. 6). These experiments were performed in both tPA^WT^ and tPA^Null^ mice. As reported above (Fig. 1A–C), tPA^Null^ mice displayed a deficit in NVC induced by whisker stimulation compared to WT animals (+ 7.16 ± 0.46% of CBF increase for WT mice, n = 18, vs. + 5.67 ± 0.49% for tPA^Null^ mice, n = 16, i.e. − 20.5% of CBF increase for tPA^Null^ mice compared to tPA^WT^ mice, p-value 0.0167), an effect also observed in WT animals following partial hepatectomy (+ 9.58 ± 0.45% for Ligatured tPA^WT^ mice, n = 9, vs. + 11.63 ± 0.63% of CBF increase for Sham tPA^WT^ mice, n = 4, i.e. − 17.5% of CBF increase for Ligatured tPA^WT^ mice compared to Sham tPA^WT^ mice, p-value 0.037). No difference was observed between tPA^WT^ and tPA^Null^ mice following partial hepatectomy (+ 10.38 ± 0.7% for Ligatured tPA^Null^ mice, n = 10, vs. + 9.58 ± 0.45% of CBF increase for Ligatured tPA^WT^ mice, n = 9, i.e. + 8.3% of CBF increase for Ligatured tPA^Null^ mice compared to Ligatured tPA^WT^ mice, p-value 0.41). The data suggest that the vascular tPA involved in NVC induced by whisker stimulation is from liver origin. To determine whether this tPA from liver originate was produced by endothelial cells or hepatocytes, we performed conditional deletion of hepatocytic tPA (see methods section) (Additional file 1: Fig. S7). Using the VECad-Cre^ΔtPA^ mice, we performed hydrodynamic transfection of hepatocytes using a pLIVE-Cre-GFP promoter. Anti-GFP and anti-CRE immunostainings confirmed the hepatocytic expression of the Cre-GFP in hepatocytes (Additional file 1: Fig. S7), leading to an additional conditional deletion (in addition to the endothelial deletion of tPA reported above (see Fig. 6) of a putative hepatocytic tPA). Our data revealed no modification of NVC in VECad-Cre^ΔtPA^ mice transfected with a pLIVE-Cre-GFP (conditional deletion of endothelial and heptacytic tPA) compared to VECad-Cre^ΔtPA^ mice transfected with an empty-pLIVE (conditional deletion of endothelial tPA only) (Additional file 1: Fig. S8) (+ 10.45 ± 0.59% of CBF increase for VECad-Cre^ΔtPA^ mice transfected with pLIVE-CRE-GFP, n = 6, vs. + 11.36 ± 0.7% for VECad-Cre^ΔtPA^ mice transfected with empty-pLive, n = 10, i.e. + 8.64% of CBF increase for VECad-Cre^ΔtPA^ mice transfected with pLIVE-CRE-GFP compared to VECad-Cre^ΔtPA^ mice transfected with an empty-pLIVE, p-value 0.29). Altogether, these data demonstrate that the tPA involved in NVC induced by whisker stimulation is from liver endothelial origin. These data are in agreement with the immunohistochemistry performed from mouse brain and liver tissues (Fig. 7), confirming the presence of tPA in both liver and brain endothelial cells.

## Discussion

We have demonstrated that physiological levels of circulating tPA, from liver origin, contribute to the modulation of functional hyperemia mediated by brain endothelial NMDAR activation. Indeed, deficiency in tPA reduced functional hyperemia induced by whisker stimulation in mice, whereas deficiency in PAI-1 increased it. Deletion of tPA was rescued by intravenous injection of tPA or by parabiosis with WT animals. Our data also reveal a key role of endothelial cells in this process, with conditional deletion of endothelial tPA leading to reduced functional hyperemia induced by whisker stimulation and deletion of endothelial NMDARs leading to a lack of response to tPA-induced NVC. We also show that bradykinin receptors type 2 activation induces both release of tPA in the circulation and subsequent potentiation of NVC. Our data also demonstrate that physiological levels of vascular tPA directly contribute to the efficiency of neurovascular coupling. Finally, we evidence that the vascular tPA involved in the NVC induced by whisker stimulation is from liver endothelial cells origin. Altogether, these data unmask a new pathway contributing to the complex regulation of neurovascular coupling.

This new mechanism may explain some of the physiological and pathological brain functions of tPA reported so far. For instance, tPA was reported to display important functions in learning and memory processes [10, 11], cognitive processes directly linked with neuronal activation and subsequent neurovascular coupling [38]. Similarly, tPA and its inhibitor PAI-1 have been reported to interfere with Aβ-induced attenuation of NVC [17]. Up to know and in agreement with an extensive literature, mechanisms of NVC associated with these phenotypes, were mainly associated with events occurring from the parenchyma to the vasculature. Our present data lead to integrate that NVC also involves mechanisms occurring from the blood stream to the vasculature [3]. It is thus interesting to note that blood transfusion from young to old mice, was reported to revitalize old brain and to reverse subsequent decline [39].

NVC has been linked to neuronal activation in general, mediated by a wide variety of vasoactive agents targeting different segments of the cerebrovascular tree [1]. Of these, Nitric Oxyde (NO) derived from NMDAR activity mediates a significant fraction of the response, and tPA is required for the full expression of this component by enabling NO production during NMDAR activation [16, 18]. Here we found that the levels of circulating tPA activity also contribute to NVC. NMDAR signaling is a key process of neuron-mediated NVC [40]. Here, we demonstrate that the NMDAR-dependent control of NVC also involves NMDARs expressed at the luminal side of endothelial cells. Endothelial NMDARs have already been identified as key actors of neuroinflammatory processes [20], also known to influence the efficiency of NVC [41].

Some studies have indicated that tPA modulates Ca^2+^ influx through NMDAR by interacting with the extracellular domain of the GluN1 subunit [42]. Other studies have suggested that tPA does not act on NMDAR directly, but through LRP1 (Low Density Lipoprotein Related Protein-1) [43–45], which, like neuronal Oxyde Nitric Synthase (nNOS), is bridged to the NMDAR complex through the adaptor protein Post Synaptic Density Protein-95 (PSD-95) [46–49]. All these studies were reported from neuronal investigations. But NMDARs are also exhibited by endothelial cells [18, 22, 50–56] and LRP1 too [57, 58]. In our hands, endothelial NMDAR were revealed on the luminal part of the vessels [20]. On endothelial cells, tPA was reported to mediate NMDAR-dependent signaling, involving differential phosphorylation of proteins associated with tight-junctions, such as the myosin light chain (MLC-1) [50]. Our observation that the effects of vascular tPA on functional hyperemia are prevented by the conditional deletion of endothelial GluN1 subunit of NMDARs are supported by the above literature. Our data also suggest that endothelial NMDAR play a negative vasoconstrictive tonus on the vessels in basal conditions, a process inhibited by tPA.

Increases in PAI-1 expression have been reported in relation with neurovascular and cardiovascular diseases [59–61], as well as in neurodegenerative disorders, especially Alzheimer’s disease [62], during which NVC is dramatically affected [63–65]. Thus, targeting the tPA-dependent and NMDAR-dependent modulation of NVC may have important clinical applications.

Bradykinin (BK) is a vasoactive polypeptide with cardioprotective effects [66], causing endothelium-dependent vasodilation by activating its endothelial B2 receptors in the lumen [67]. Interestingly, BK has been shown to promote tPA secretion in isolated perfused vascular systems [68]. Here, we evidenced that BK, and not angiotensin, promotes the release of active tPA in the blood stream in vivo via B2R, leading to a tPA-dependent specific increase in NVC. PAI-1 is among the factors released in the circulation by endothelial cells in the context of endothelial dysfunction [69]. At the opposite release of tPA is more usually associated with normal endothelial function [70, 71]. In parallel, it is widely accepted that vascular endothelial cells, through the release of NO, prostacyclin, tPA and PAI-1, play important roles in the regulation of thrombosis and fibrinolysis, mechanisms also highly related with impaired NVC [72–74].

The consensus would be that the majority of tPA in the blood stream would come from hepatocytes and that endothelial cells would contribute to the local and/or fast changes in tPA levels [75, 76]. Our present data reveal that the vascular tPA involved in the control of NVC induced by whisker stimulation indeed comes from the liver, but from endothelial origin.

In conclusion, we have demonstrated that changes in circulating tPA levels, modulated by activation of endothelial bradykinin receptors, presumably coming from the liver endothelial cells, tunes NVC, through the modulation of cerebral endothelial NMDARs. The data unveil a previously unknown role for circulating tPA in the control of neurovascular coupling and subsequent cerebral physiopathology axis with potential therapeutic implications.

## Supplementary Information

**Additional file 1: Figure S1.** Recording of physiological parameters during whisker stimulations. Mice were mechanically ventilated (120 BPM, 10 ml/kg). Blood samples were collected by a femoral catheter and mean arterial pressure (MAP) was measured. Pharmacological treatments were injected via a tail vein catheter. Data are mean with SD, n=8. **Figure S2.** Parabiosis model and chimerism validation methods. **A**: Schematic representation of experimental study design for parabiosis surgery and chimerism validation. **B-D**: Parabiosis chimerism validation by IV injection of glucose (100µl, 1.2g/kg) at T0 in tail vein of donor mice, glycemia (mg/dl) of both donor and recipient mice was measured during 1 hour, at different time points following parabiosis surgery (D5, n=5, figure **B**; D15, n=7, figure **C**; D22, n=7, figure **D**). **E**: Representative MRI acquisition of parabionts after IV DOTA-gd injection (200µl, 0.01mMol/ml) in donor mice to validate parabiosis model. T1-weighted image (red) represents DOTA- Gd signal and was superposed on anatomical T2-weighted image. **F**: Quantification of DOTA-Gd distribution between parabionts at different time points. Data are mean with SD for each group. *p < 0.05, **p < 0.005 and ***p < 0.001 from T0, 2-way ANOVA test. **Figure S3.** Mean arterial blood pressure and blood gases before and after injection of B2Rag. **A**: Mean arterial pressure (MAP, mmHg) before (-B2Rag) and after IV injection of 30 µg/kg of B2Rag (+B2Rag). Data are Mean with values for each mouse. **B-C**: Measurement of arterial blood gas before (-B2Rag, **C**) and after IV injection of 30 µg/kg of B2Rag (+B2Rag, **D**). pCO2, pO2, O2sat and pH were measured with 100µl of blood on RAPIDLab^®^ 348EX Blood Gas System. Data are mean with values for each mouse and reference range for these measurements. *p < 0.05 from - B2Rag, Mann-Whitney test, n=4 per group. **Figure S4.** Partial hepatectomy does not influence arterial blood pressure. Mean arterial pressure (MAP, mmHg) before and 24h after hepatectomy in tPA^WT^ mice. Data are Mean with values for each mouse. Mann-Whitney test, n=5 per group. **Figure S5.** Mean arterial blood pressure and blood gases before and after injection of Ang-II in mice. **A**: Mean arterial pressure (MAP, mmHg) before (-Ang-II) and 25 minutes after IV infusion of 1 µg/kg/min of Ang-II (+Ang-II). Data are Mean with values for each mouse. **B-C**: Measurement of arterial blood gas before (-Ang-II, **C**) and 25 minutes after IV infusion of 1 µg/kg/min of Ang-II (+Ang-II, **D**). pCO2, pO2, O2sat and pH were measured with 100µl of blood on RAPIDLab® 348EX Blood Gas System. Data are mean with values for each mouse and reference range for these measurements. *p < 0.05 from -Ang-II, Mann-Whitney test, n=4 per group. **Figure S6.** Intravenous administration of angiotensin-II (Ang-II) does not influence NVC in a tPA dependent manner. **Figure S7.** Hydrodynamic transfection of pLIVE-Cre-GFP in VECad-CreΔtPA mice. **A**: Construction scheme of plasmid pLIVE-Cre-GFP. The cDNA of Cre-GFP was PCR amplified from a pCAG-Cre-GFP plasmid and fused into multiple cloning sites of the pLIVE plasmid between BamHI and NotI. **B**: Experimental schematic representation of hydrodynamic transfection of empty-pLIVE or pLIVE-Cre-GFP. 48h after transfection of the plasmid, livers from transfected VECad-CreΔtPA mice were collected. **C**: Epifluorescence images of liver slices from transfected VECad-CreΔtPA mice with empty-pLIVE or pLIVE- Cre-GFP (scale bar = 500µm). Immunostaining reveals GFP (green), Cre-recombinase (red) and actin filaments with phalloidin (grey), confirming the transfection of the plasmid in hepatocytes. Images were digitally captured using a camera (CoolSNAP; Photometrics) and/or with an inverted confocal microscope (SP5, Leica). Images werevisualized respectively with Metavue 5.0 software (Molecular Devices, USA) and LAS AF lite software (LEICA). **Figure S8.** Deletion of tPA in hepatocytes does not influence NVC in VECad-Cre^ΔtPA^ mice. **Figure S9.** tPA expression in wild type (tPA^WT^) and tPA deficient mice (tPA^Null^). **A**: Representative photomicrographs (n=5) of hippocampal tissue sections subjected to immunostainings raised against tPA (red), CD31 (green) and DAPI staining (blue); scale bar 100µm and 50µm in the magnified inserts. **B**: Quantification of tPA fluorescent intensity in hippocampus of tPA^WT^ and tPA^Null^ mice. **C**: Representative photomicrographs of hippocampal tissue sections with *plat *(gene encoding for tPA; green) revealed by *in situ* hybridization (ISH), immunostaining raised against CD31 (red) and DAPI (blue) staining; scale bar 100µm. **D**: Quantifications of *plat *positive cells in the hippocampus of tPA^WT^ (N=1; n=30) and tPA^Null^ mice (N=1; n=30). **Figure S10.** tPA expression in wild type (tPA^WT^) and tPA deficient mice (tPA^Null^). **A**: Representative photomicrographs of hippocampal tissue sections with *plat *(*in situ* hybridization for the mRNA encoding tPA; green) and neurons labelled using neurotrace (red) stainings; scale bar 100µm. **B**: Quantifications of *plat *positive cells in hippocampus in tPA^WT^ (N=1; n=85) and tPA^Null^ (N=1; n=40) mice. **C**: Representative photomicrographs of cortical tissue sections with *plat* (*in situ* hybridization for the mRNA encoding tPA; green) and neurons labelled using neurotrace (red) stainings; scale bar 100µm. **D**: Quantifications of *plat *positive cells in the cortex of tPA^WT^ (N=1; n=100) and tPA^Null^ (N=1; n=40) mice. **Figure S11.** Conditional deletion of GluN1 in VeCad-Cre conditional knockout mice. **A**: Representative photomicrographs (n=5) of hippocampal tissue sections for *grin1* (*in situ* hybridization of the gene encoding for the GluN1 subunit of NMDA receptor; green), immunostainings against CD31 (red) and DAPI (blue) staining; scale bar 100µm. **B**: Quantifications of *grin1 *positive cells in VEcad-Cre^WT^ (N=1; n=30) and VEcad-Cre ^Δ^^GluN1^ (N=1; n=30) mice. **Figure S12.** Conditional deletion of tPA in VeCad-Cre conditional knockout mice. **A**: Representative photomicrographs (n=5) of hippocampal tissue sections for *plat *(*in situ* hybridization of the gene encoding for tPA; green), immunostainings against CD31 (red) and DAPI (blue) staining; scale bar 100µm. **B**: Quantifications of *plat *positive cells in VEcad-Cre^WT^ (N=1; n=25) and VEcad-Cre^Δ^^tPA^ (N=1; n=30) mice.

## Data Availability

The datasets analyzed during the current study are available from the corresponding author on request.

## Abbreviations

Ang-II: Angiotensin-II
B2Rag: Selective bradykinin type 2 receptor agonist
BK: Bradykinin
CBF: Cerebral blood flow
CNS: Central nervous system
MAP: Mean arterial pressure
MLC: Myosin light chain
NMDAR: N-methyl-d-aspartate receptors
NO: Nitric oxide
NVC: Neurovascular coupling
PAI-1: Type 1 Plasminogen Activator Inhibitor
PSD-95: Post Synaptic Density Protein-95
rtPA: Recombinant tPA
tPA: Tissue-type plasminogen activator

## References

[1] Iadecola C (2017). The Neurovascular unit coming of age: a journey through neurovascular coupling in health and disease. Cell Press: Neuron.

[2] Sweeney MD, Kisler K, Montagne A, Toga AW, Zlokovic B (2018). The role of brain vasculature in neurodegenerative disorders. Nat Neurosci.

[3] Girouard H, Iadecola C (2006). Neurovascular coupling in the normal brain and in hypertension, stroke, and Alzheimer disease. J Appl Physiol.

[4] Collen D, Lijnen HR (2004). Tissue-type plasminogen activator: a historical perspective and personal account. J Thromb Haemost.

[5] Teesalu T, Kulla A, Simisker A, Sirén V, Lawrence DA, Asser T (2004). Tissue plasminogen activator and neuroserpin are widely expressed in the human central nervous system. Thromb Haemost.

[6] Louessard M, Lacroix A, Martineau M, Mondielli G, Montagne A, Lesept F (2016). Tissue plasminogen activator expression is restricted to subsets of excitatory pyramidal glutamatergic neurons. Mol Neurobiol.

[7] Lochner JE, Honigman LS, Grant WF, Gessford SK, Hansen AB, Silverman MA (2006). Activity-dependent release of tissue plasminogen activator from the dendritic spines of hippocampal neurons revealed by live-cell imaging. J Neurobiol.

[8] Angles-Cano E, Balaton A, le Bonniec B, Genot E, Elion J, Sultan Y (1985). Production of monoclonal antibodies to the high fibrin-affinity, tissue- type plasminogen activator of human plasma. Demonstration of its endothelial origin by immunolocalization. Blood.

[9] Thiebaut AM, Gauberti M, Ali C, Martinez de Lizarrondo S, Vivien D, Yepes M (2018). The role of plasminogen activators in stroke treatment: fibrinolysis and beyond. Lancet Neurol.

[10] Hébert M, Anfray A, Chevilley A, Martinez de Lizarrondo S, Quenault A, Louessard M (2017). Distant space processing is controlled by tPA-dependent NMDA receptor signaling in the entorhinal cortex. Cereb Cortex.

[11] Benchenane K, Castel H, Boulouard M, Bluthé R, Fernandez-Monreal M, Roussel BD (2007). Anti-NR1 N-terminal-domain vaccination unmasks the crucial action of tPA on NMDA-receptor-mediated toxicity and spatial memory. J Cell Sci.

[12] Pawlak R, Magarinos AM, Melchor J, McEwen B, Strickland S (2003). Tissue plasminogen activator in the amygdala is critical for stress-induced anxiety-like behavior. Nat Neurosci.

[13] Matys T, Pawlak R, Matys E, Pavlides C, McEwen BS, Strickland S (2004). Tissue plasminogen activator promotes the effects of corticotropin-releasing factor on the amygdala and anxiety-like behavior. Proc Natl Acad Sci USA.

[14] Wardlaw JM, Doubal FN, Valdes-Hernandez M, Wang X, Chappell FM, Shuler K (2013). Blood-brain barrier permeability and long-term clinical and imaging outcomes in cerebral small vessel disease. Stroke.

[15] Marcos-Contreras OA, Martinez de Lizarrondo S, Bardou I, Orset C, Pruvost M, Anfray A (2016). Hyperfibrinolysis increases blood-brain barrier permeability by a plasmin-and bradykinin-dependent mechanism. Blood.

[16] Park L, Gallo EF, Anrather J, Wang G, Norris EH, Paul J (2008). Key role of tissue plasminogen activator in neurovascular coupling. Proc Natl Acad Sci USA.

[17] Park L, Zhou J, Koizumi K, Wang G, Anfray A, Ahn SJ (2020). tPA deficiency underlies neurovascular coupling dysfunction by amyloid-b. J Neurosci.

[18] Anfray A, Drieu A, Hingot V, Hommet Y, Yetim M, Rubio M (2020). Circulating tPA contributes to neurovascular coupling by a mechanism involving the endothelial NMDA receptors. J Cereb Blood Flow Metab.

[19] Nakazawa K, McHugh TJ, Wilson MA, Tonegawa S (2004). NMDA receptors, place cells and hippocampal spatial memory. Nat Rev Neurosci.

[20] Macrez R, Ortega MC, Bardou I, Mehra A, Fournier A, van der Pol SMA (2016). Neuroendothelial NMDA receptors as therapeutic targets in experimental autoimmune encephalomyelitis. Brain.

[21] Reijerkerk A, Kooij G, van der Pol SMA, Leyen T, Lakeman K, van het Hof B (2010). The NR1 subunit of NMDA receptor regulates monocyte transmigration through the brain endothelial cell barrier. J Neurochem.

[22] Peters EC, Gee MT, Pawlowski LN, Kath AM, Polk FD, Vance CJ (2022). Amyloid-β disrupts unitary calcium entry through endothelial NMDA receptors in mouse cerebral arteries. J Cereb Blood Flow Metab.

[23] Zheng Z, Nayak L, Wang W, Yurdagul A, Wang X, Cai B (2019). An ATF6-tPA pathway in hepatocytes contributes to systemic fibrinolysis and is repressed by DACH1. Blood.

[24] Samad F, Yamamoto K, Loskutoff DJ (1996). Distribution and regulation of plasminogen activator inhibitor-1 in murine adipose tissue in vivo: induction by tumor necrosis factor-α and lipopolysaccharide. J Clin Investig.

[25] Ny T, Sawdey M, Lawrence D, Millan JL, Loskutoff DJ (1986). Cloning and sequence of a cDNA coding for the human beta-migrating endothelial-cell-type plasminogen activator inhibitor. Proc Natl Acad Sci USA.

[26] Medcalf RL (2017). Fibrinolysis: from blood to the brain. J Thromb Haemost.

[27] Shi K, Zou M, Jia DM, Shi S, Yang X, Liu Q (2021). tPA Mobilizes immune cells that exacerbate hemorrhagic transformation in stroke. Circ Res.

[28] Brown NJ, Gainer JV, Murphey LJ, Vaughan DE (2000). Bradykinin stimulates tissue plasminogen activator release from human forearm vasculature through B(2) receptor-dependent, NO synthase-independent, and cyclooxygenase-independent pathway. Circulation.

[29] Kerins DM, Hao Q, Vaughan DE (1995). Angiotensin induction of PAI-1 expression in endothelial cells is mediated by the hexapeptide angiotensin IV. J Clin Investig.

[30] Nordt TK, Lohrmann J, Bode C (2001). Regulation of PAI-1 expression by genetic polymorphisms. Impact on atherogenesis. Thromb Res.

[31] Pasquet N, Douceau S, Naveau M, Lesept F, Louessard M, Lebouvier L (2019). Tissue-type plasminogen activator controlled corticogenesis through a mechanism dependent of NMDA receptors expressed on radial glial cells. Cereb Cortex.

[32] Léger C, Dupré N, Aligny C, Bénard M, Lebon A, Henry V (2020). Glutamate controls vessel-associated migration of GABA interneurons from the pial migratory route via NMDA receptors and endothelial protease activation. Cell Mol Life Sci.

[33] Alva JA, Zovein AC, Monvoisin A, Murphy T, Salazar A, Harvey NL (2006). VE-cadherin-cre-recombinase transgenic mouse: a tool for lineage analysis and gene deletion in endothelial cells. Dev Dyn.

[34] Kamran P, Sereti KI, Zhao P, Ali SR, Weissman IL, Ardehali R (2013). Parabiosis in mice: a detailed protocol. J Vis Exp.

[35] Vaughan DE, Lazos SA, Tong K (1995). Angiotensin II regulates the expression of plasminogen activator inhibitor-1 in cultured endothelial cells: a potential link between the renin-angiotensin system and thrombosis. J Clin Investig.

[36] Minai K, Matsumoto T, Horie H, Ohira N, Takashima H, Yokohama H (2001). Bradykinin stimulates the release of tissue plasminogen activator in human coronary circulation: effects of angiotensin-converting enzyme inhibitors. J Am Coll Cardiol.

[37] Ping Zhang L, Takahara T, Yata Y, Furui K, Jin B, Kawada N (1999). Increased expression of phninogen activator and phsminogen activator inhibitor during liver fibrogenesis of ratx role of stellate cells. J Hepatol.

[38] Lefferts WK, Deblois JP, Barreira TV, Heffernan KS (2018). Neurovascular coupling during cognitive activity in adults with controlled hypertension. J Appl Physiol.

[39] Wyss-Coray T (2016). Ageing, neurodegeneration and brain rejuvenation. Nature.

[40] Mapelli L, Gagliano G, Soda T, Laforenza U, Moccia F, D’Angelo EU (2017). Granular layer neurons control cerebellar neurovascular coupling through an NMDA receptor/NO-dependent system. J Neurosci.

[41] Fulop GA, Ahire C, Csipo T, Tarantini S, Kiss T, Balasubramanian P (2019). Cerebral venous congestion promotes blood-brain barrier disruption and neuroinflammation, impairing cognitive function in mice. Geroscience.

[42] Nicole O, Docagne F, Ali C, Margaill I, Carmeliet P, MacKenzie ET (2001). The proteolytic activity of tissue-plasminogen activator enhances NMDA receptor-mediated signaling. Nat Med.

[43] Martin AM, Kuhlmann C, Trossbach S, Jaeger S, Waldron E, Roebroek A (2008). The functional role of the second NPXY motif of the LRP1 β-chain in tissue-type plasminogen activator-mediated activation of N-methyl-D-aspartate receptors. J Biol Chem.

[44] Samson AL, Nevin ST, Croucher D, Niego B, Daniel PB, Weiss TW (2008). Tissue-type plasminogen activator requires a co-receptor to enhance NMDA receptor function. J Neurochem.

[45] Mantuano E, Lam MS, Gonias SL (2013). LRP1 assembles unique co-receptor systems to initiate cell signaling in response to tissue-type plasminogen activator and myelin-associated glycoprotein. J Biol Chem.

[46] Gotthardt M, Trommsdorff M, Nevitt MF, Shelton J, Richardson JA, Stockinger W (2000). Interactions of the low-density lipoprotein receptor gene family with cytosolic adaptor and scaffold proteins suggest diverse biological functions in cellular communication and signal transduction. J Biol Chem.

[47] May P, Rohlmann A, Bock HH, Zurhove K, Marth JD, Schomburg ED (2004). Neuronal LRP1 functionally associates with postsynaptic proteins and is required for normal motor function in mice. Mol Cell Biol.

[48] Nakajima C, Kulik A, Frotscher M, Herz J, Schäfer M, Bock HH (2013). Low-density lipoprotein receptor-related protein 1 (LRP1) modulates N-methyl-D-aspartate (NMDA) receptor-dependent intracellular signaling and NMDA-induced regulation of postsynaptic protein complexes. J Biol Chem.

[49] Norris EH, Strickland S (2007). Modulation of NR2B-regulated contextual fear in the hippocampus by the tissue plasminogen activator system. Proc Natl Acad Sci USA.

[50] Mehra A, Guérit S, Macrez R, Gosselet F, Sevin E, Lebas H (2020). Nonionotropic action of endothelial NMDA receptors on blood–brain barrier permeability via Rho/ROCK-mediated phosphorylation of myosin. J Neurosci.

[51] Kim KS, Jeon MT, Kim ES, Lee CH, Kim DG (2022). Activation of NMDA receptors in brain endothelial cells increases transcellular permeability. Fluids Barriers of CNS.

[52] Epping L, Schroeter C, Nelke C, Bock S, Gola L, Ritter N (2022). Activation of non-classical NMDA receptors by glycine impairs barrier function of brain endothelial cells. Cell Mol Life Sci.

[53] Stanton JA, Williams EI, Betterton RD, Davis TP, Ronaldson PT (2022). Targeting organic cation transporters at the blood-brain barrier to treat ischemic stroke in rats. Exp Neurol.

[54] Fournier AP, Tastet O, Charabati M, Hoornaert C, Bourbonnière L, Klement W (2022). Single-cell transcriptomics identifies brain endothelium inflammatory networks in experimental autoimmune encephalomyelitis. Neurol Neuroimmunol Neuroinflamm.

[55] Sabbagh MF, Heng JS, Luo C, Castanon RG, Nery JR, Rattner A (2018). Transcriptional and epigenomic landscapes of CNS and non-CNS vascular endothelial cells. Elife.

[56] Yang AC, Vest RT, Kern F, Lee DP, Agam M, Maat CA (2022). A human brain vascular atlas reveals diverse mediators of Alzheimer's risk. Nature.

[57] Shibata M, Yamada S, Ram Kumar S, Calero M, Bading J, Frangione B (2000). Clearance of Alzheimer’s amyloid-β1-40 peptide from brain by LDL receptor-related protein-1 at the blood-brain barrier. J Clin Investig.

[58] Storck SE, Pietrzik CU (2017). Endothelial LRP1—a potential target for the treatment of Alzheimer’s Disease: theme: drug discovery, development and delivery in alzheimer’s disease guest editor: Davide Brambilla. Pharm Res.

[59] Jung RG, Simard T, Labinaz A, Ramirez FD, di Santo P, Motazedian P (2018). Role of plasminogen activator inhibitor-1 in coronary pathophysiology. Thromb Res.

[60] Yu BY, Subudeng G, Du CG, Liu ZH, Zhao YF, Namei E (2019). Plasminogen activator, tissue type regulates germinal vesicle breakdown and cumulus expansion of bovine cumulus-oocyte complex in vitro. Biol Reprod.

[61] Rosenberg RD, Aird WC (1999). Vascular-bed–specific hemostasis and hypercoagulable states. N Engl J Med.

[62] Oh J, Lee HJ, Song JH, Park SI, Kim H (2014). Plasminogen activator inhibitor-1 as an early potential diagnostic marker for Alzheimer’s disease. Exp Gerontol.

[63] Tarantini S, Tran CHT, Gordon GR, Ungvari Z, Csiszar A (2017). Impaired neurovascular coupling in aging and Alzheimer’s disease: contribution of astrocyte dysfunction and endothelial impairment to cognitive decline. Exp Gerontol.

[64] Nicolakakis N, Aboulkassim T, Ongali B, Lecrux C, Fernandes P, Rosa-Neto P (2008). Complete rescue of cerebrovascular function in aged Alzheimer’s disease transgenic mice by antioxidants and pioglitazone, a peroxisome proliferator-activated receptor γ agonist. J Neurosci.

[65] Kisler K, Nelson AR, Montagne A, Zlokovic BV (2017). Cerebral blood flow regulation and neurovascular dysfunction in Alzheimer disease. Nat Rev Neurosci.

[66] Starkopf J, Bugge E, Ytrehus K (1997). Preischemic bradykinin and ischaemic preconditioning in functional recovery of the globally ischaemic rat heart. Cardiovasc Res.

[67] van Guilder GP, Pretorius M, Luther JM, Byrd JB, Hill K, Gainer JV (2008). Bradykinin type 2 receptor BE1 genotype influences bradykinin-dependent vasodilation during angiotensin-converting enzyme inhibition. Hypertension.

[68] Emeis JJ (1983). Perfused rat hindlegs. A model to study plasminogen activator release. Thromb Res.

[69] Jacobs A, Schutte AE, Ricci C, Pieters M (2019). Plasminogen activator inhibitor-1 activity and the 4G/5G polymorphism are prospectively associated with blood pressure and hypertension status. J Hypertens.

[70] Stein CM, Brown N, Vaughan DE, Lang CC, Wood AJ (1998). Regulation of local tissue-type plasminogen activator release by endothelium-dependent and endothelium-independent agonists in human vasculature. J Am Coll Cardiol.

[71] Muldowney JA, Vaughan DE (2002). Tissue-type plasminogen activator release: new frontiers in endothelial function. J Am Coll Cardiol.

[72] Thors B, Halldórsson H, Jónsdóttir G, Thorgeirsson G (2008). Mechanism of thrombin mediated eNOS phosphorylation in endothelial cells is dependent on ATP levels after stimulation. Biochim Biophys Acta.

[73] Koide M, Bonev AD, Nelson MT, Wellman GC (2012). Inversion of neurovascular coupling by subarachnoid blood depends on large-conductance Ca2+-activated K+(BK) channels. Proc Natl Acad Sci USA.

[74] Braaten JV, Handt S, Jerome WG, Kirkpatrick J, Lewis JC, Hantgan RR (1993). Regulation of fibrinolysis by platelet-released plasminogen activator inhibitor 1: light scattering and ultrastructural examination of lysis of a model platelet-fibrin thrombus. Blood.

[75] Zheng Z, Nakamura K, Gershbaum S, Wang X, Thomas S, Bessler M (2020). Interacting hepatic PAI-1/tPA gene regulatory pathways influence impaired fibrinolysis severity in obesity. J Clin Investig.

[76] Emeis J (1995). The Control of tPA and PAI-1 Secretion from the Vessel Wall. Vasc Med Rev.

