# Supplementary material for: Blood tissue Plasminogen Activator (tPA) of liver origin contributes to neurovascular coupling involving brain endothelial N-Methyl-D-Aspartate (NMDA) receptors
Source: Fluids Barriers CNS. 2023 Feb 3;20:11. doi: 10.1186/s12987-023-00411-w (PMC9896721; doi:10.1186/s12987-023-00411-w)
Supplement: Supplementary file 1 — Additional file 1: Figure S1. Recording of physiological parameters during whisker stimulations. Mice were mechanically ventilated (120 BPM, 10 ml/kg). Blood samples were collected by a femoral catheter and mean arterial pressure (MAP) was measured. Pharmacological treatments were injected via a tail vein catheter. Data are mean with SD, n=8. Figure S2. Parabiosis model and chimerism validation methods. A: Schematic representation of experimental study design for parabiosis surgery and chimerism validation. B-D: Parabiosis chimerism validation by IV injection of glucose (100µl, 1.2g/kg) at T0 in tail vein of donor mice, glycemia (mg/dl) of both donor and recipient mice was measured during 1 hour, at different time points following parabiosis surgery (D5, n=5, figure B; D15, n=7, figure C; D22, n=7, figure D). E: Representative MRI acquisition of parabionts after IV DOTA-gd injection (200µl, 0.01mMol/ml) in donor mice to validate parabiosis model. T1-weighted image (red) represents DOTA- Gd signal and was superposed on anatomical T2-weighted image. F: Quantification of DOTA-Gd distribution between parabionts at different time points. Data are mean with SD for each group. *p < 0.05, **p < 0.005 and ***p < 0.001 from T0, 2-way ANOVA test. Figure S3. Mean arterial blood pressure and blood gases before and after injection of B2Rag. A: Mean arterial pressure (MAP, mmHg) before (-B2Rag) and after IV injection of 30 µg/kg of B2Rag (+B2Rag). Data are Mean with values for each mouse. B-C: Measurement of arterial blood gas before (-B2Rag, C) and after IV injection of 30 µg/kg of B2Rag (+B2Rag, D). pCO2, pO2, O2sat and pH were measured with 100µl of blood on RAPIDLab® 348EX Blood Gas System. Data are mean with values for each mouse and reference range for these measurements. *p < 0.05 from - B2Rag, Mann-Whitney test, n=4 per group. Figure S4. Partial hepatectomy does not influence arterial blood pressure. Mean arterial pressure (MAP, mmHg) before and 24h after hepatectomy in tP [file 12987_2023_411_MOESM1_ESM.docx]

# Additional materials and methods

**Magnetic Resonance Imaging (MRI)**

The MRI studies were realized 3 weeks after parabiosis surgery. Parabionts were placed in a rat cradle (to image both animals at the same time). Animals were anesthetized using 5% isoflurane (Isoflurane Belamont) in 50% N2O/50% O2, then maintained under 2% isoflurane in 100% N2O/100% O2. They were monitored by a breathing module in order to adapt the anesthesia according to the respiratory rate and they were thermoregulated by a heating blanket beeween 36.5°C and 37.5°C. The chimerism validation was assessed by injecting DOTA‐Gd (Dotarem®), 0.01mMol/ml through a tail vein catheter in MRI. Then MRI sequences (7T MRI, Bruker, Germany) of T2-weighted and T1-weighted are respectively realized on whole animals’ body. MRI analyses were performed until 75 minutes after the injection. Image analyses were made with an extension of ImageJ (Fiji). We obtained final images by making substraction between “After Injection Time” and “Before Injection Time”, to correct the signal difference for T1-weighted sequence.

# Hydrodynamic transfection

Hydrodynamic transfections were performed as previously described34. Awake mice were injected with either 100µg engineered pLIVE encoding Cre-green fluorescent protein (pLIVE-Cre-GFP) or pLIVE plasmid alone (empty-pLIVE). A large volume (10% of body weight) of plasmid-containing saline buffer (0.9% NaCl) was injected into the caudal vein in less than 5 seconds for hepatic transfection. After 48 hours, Laser Doppler Speckle was realized (see Laser Doppler Speckle section) to measure modifications of the CBF induced by whisker stimulations. Liver tissues were collected

after transcardiac perfusions with cold heparinized saline (15 ml/min) followed by a solution of 4% paraformaldehyde.

# Immunohistochemistry

Deeply anesthetized mice were transcardially perfused with cold heparinized saline (15 ml/min), then with 4% paraformaldehyde in 0.1 M sodium phosphate buffer, pH 7.4 (150 ml, 15 ml/min). Liver segments were removed and washed in veronal buffer containing 20% sucrose for 24h, and frozen in Tissue-Tek (Miles Scientific). Transversal sections of liver (10 µm) were cut on a cryostat, then collected on poly-D- lysine slides and stored at -80°C. Liver sections were incubated overnight at room temperature with primary antibodies rabbit anti GFP (1:1000, ab6556, Abcam), mouse anti Cre-recombinase (1:1000, MAB3120, Millipore) and phalloidin (1:1000, ab176759, Abcam). Primary antibodies were revelated using Fab’2 fragment anti-rabbit IgG linked to FITC and anti-mouse linked to CY3 (1:800, Jackson ImmunoReasearch) co- incubated 90 min at room temperature. Sections were then coverslipped using mounting medium containing DAPI. Images were digitally captured using an epifluorescence microscope (Leica DM6000). Images were assessed using ImageJ software (NIH).


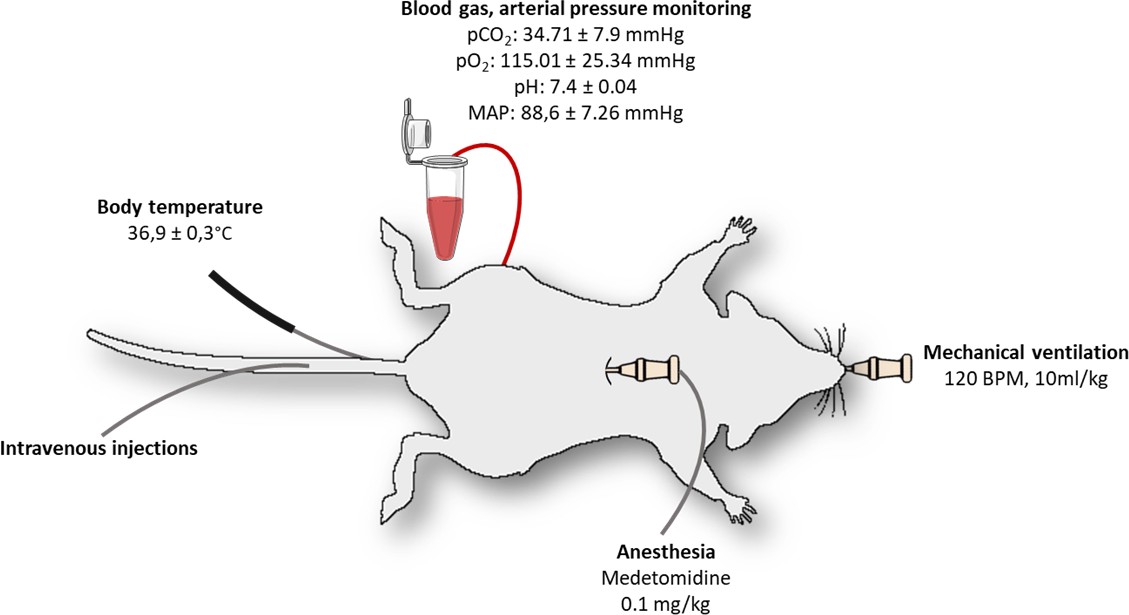


**Figure S1: Recording of physiological parameters during whisker stimulations.** Mice were mechanically ventilated (120 BPM, 10 ml/kg). Blood samples were collected by a femoral catheter and mean arterial pressure (MAP) was measured. Pharmacological treatments were injected via a tail vein catheter. Data are mean with SD, n=8.


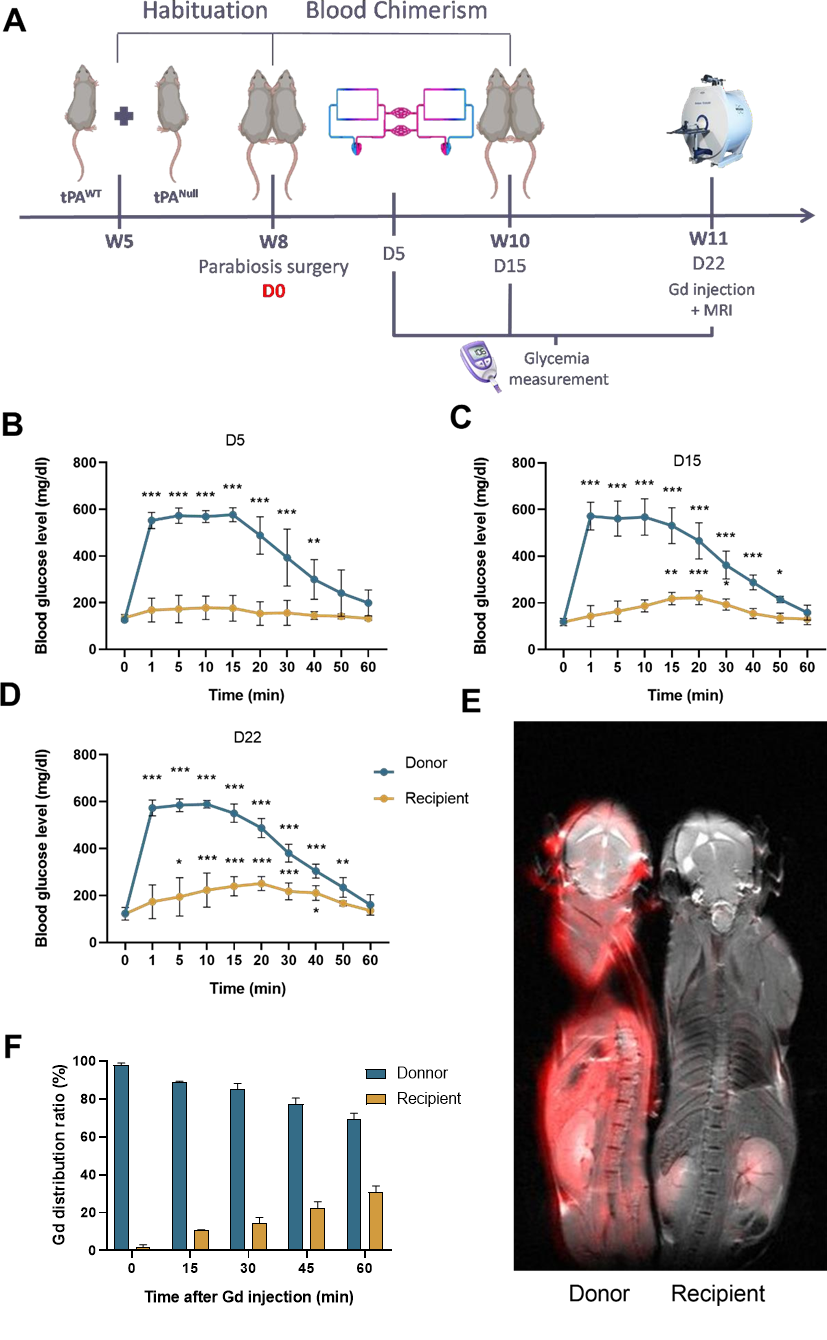


**Figure S2: Parabiosis model and chimerism validation methods. A**: Schematic representation of experimental study design for parabiosis surgery and chimerism validation. **B-D**: Parabiosis chimerism validation by IV injection of glucose (100µl, 1.2g/kg) at T0 in tail vein of donor mice, glycemia (mg/dl) of both donor and recipient mice was measured during 1 hour, at different time points following parabiosis surgery (D5, n=5, figure **B**; D15, n=7, figure **C**; D22, n=7, figure **D**). **E**: Representative MRI acquisition of parabionts after IV DOTA-gd injection (200µl, 0.01mMol/ml) in donor mice to validate parabiosis model. T1-weighted image (red) represents DOTA- Gd signal and was superposed on anatomical T2-weighted image. **F**: Quantification of DOTA-Gd distribution between parabionts at different time points. Data are mean with SD for each group. *p < 0.05, **p < 0.005 and ***p < 0.001 from T0, 2-way ANOVA test.


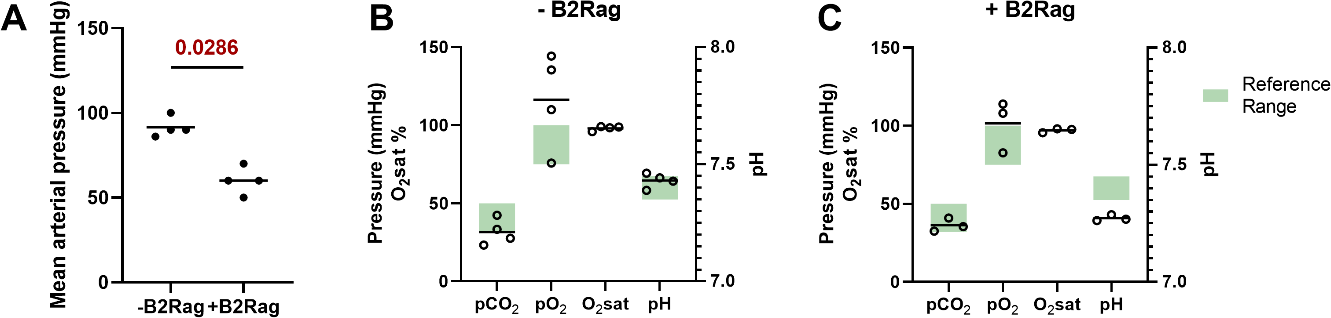


**Figure S3: Mean arterial blood pressure and blood gases before and after injection of B2Rag. A**: Mean arterial pressure (MAP, mmHg) before (-B2Rag) and after IV injection of 30 µg/kg of B2Rag (+B2Rag). Data are Mean with values for each mouse. **B-C**: Measurement of arterial blood gas before (-B2Rag, **C**) and after IV injection of 30 µg/kg of B2Rag (+B2Rag, **D**). pCO2, pO2, O2sat and pH were measured with 100µl of blood on RAPIDLab® 348EX Blood Gas System. Data are mean with values for each mouse and reference range for these measurements. *p < 0.05 from - B2Rag, Mann-Whitney test, n=4 per group.

**Figure S4: Partial hepatectomy does not influence arterial blood pressure.** Mean arterial pressure (MAP, mmHg) before and 24h after hepatectomy in tPA^WT^ mice. Data are Mean with values for each mouse. Mann-Whitney test, n=5 per group.


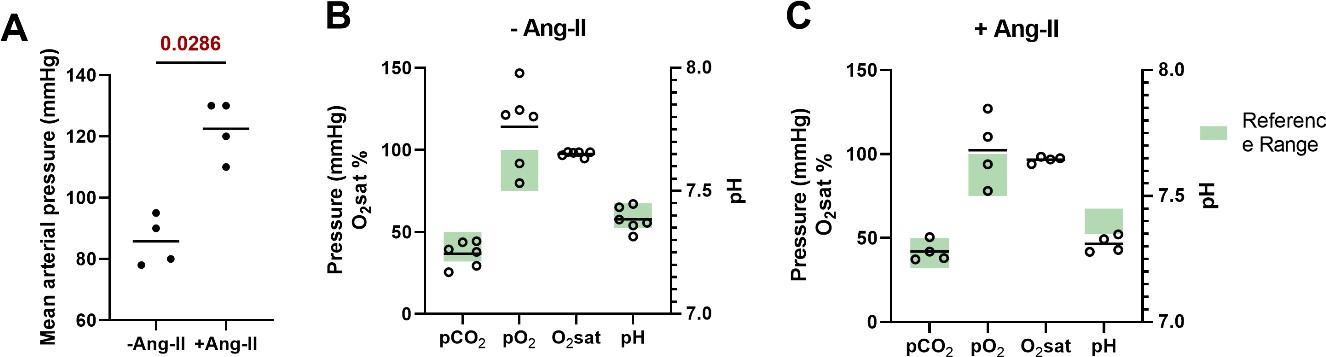


**Figure S5: Mean arterial blood pressure and blood gases before and after injection of Ang-II in mice. A**: Mean arterial pressure (MAP, mmHg) before (-Ang-II) and 25 minutes after IV infusion of 1 µg/kg/min of Ang-II (+Ang-II). Data are Mean with values for each mouse. **B-C**: Measurement of arterial blood gas before (-Ang-II, **C**) and 25 minutes after IV infusion of 1 µg/kg/min of Ang-II (+Ang-II, **D**). pCO2, pO2, O2sat and pH were measured with 100µl of blood on RAPIDLab® 348EX Blood Gas System. Data are mean with values for each mouse and reference range for these measurements. *p < 0.05 from -Ang-II, Mann-Whitney test, n=4 per group.


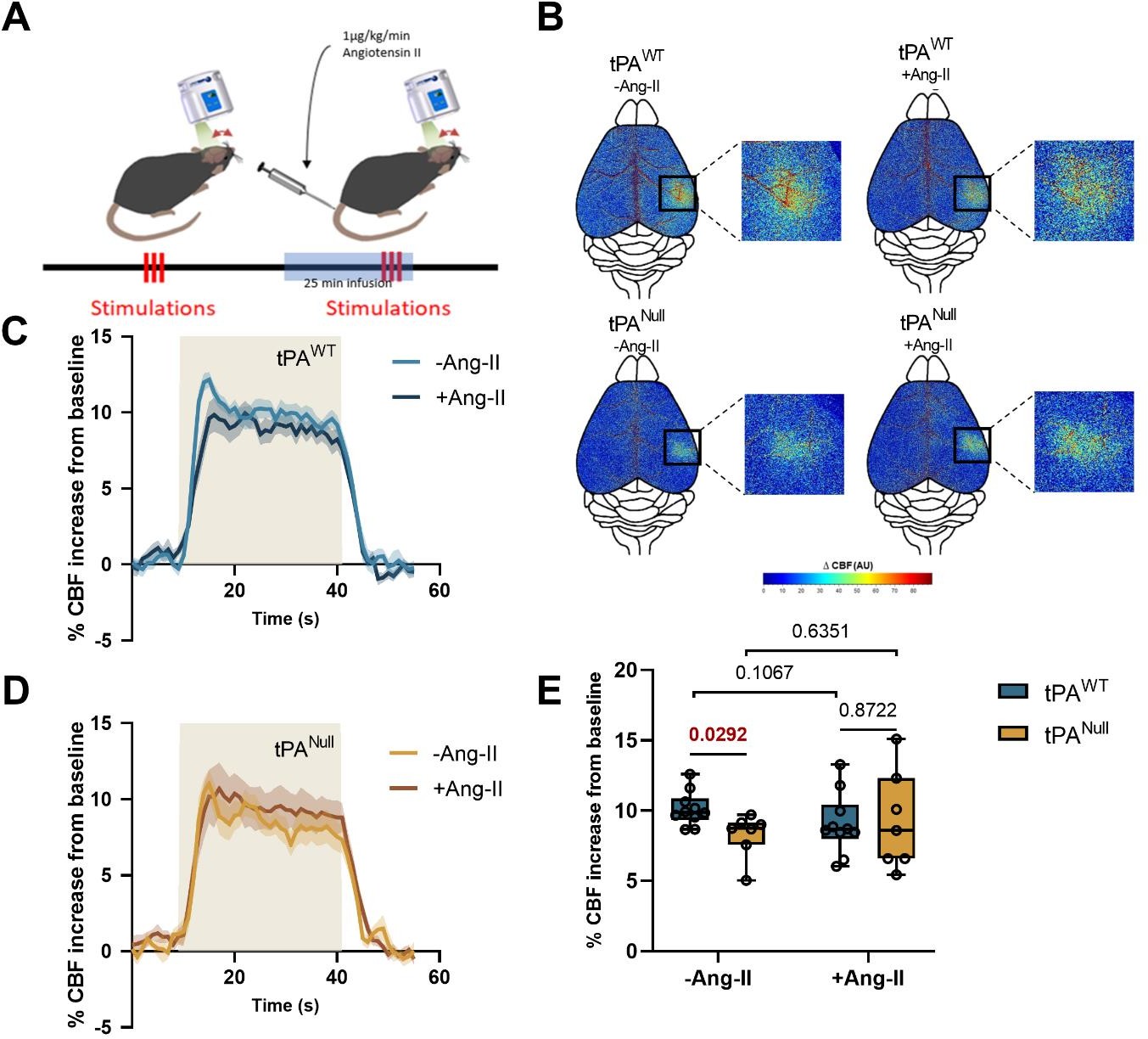


**Figure S6: Intravenous administration of angiotensin-II (Ang-II) does not influence NVC in a tPA dependent manner.** **A**: Schematic representation of the experimental timeline of whiskers stimulations paradigm. 3 trains of stimulations were made on laser speckle flowmetry before IV infusion of angiotensin-II (1 µg/kg/min) during 25 minutes, followed by 3 additonal trains of stimulations. **B**: Colormap corresponds to the activation map related ∆CBF changes during whiskers stimulations in tPANull mice and their littermate before and after IV infusion of Ang-II. Warm colours indicate an elevation of CBF during whiskers stimulations. **C-D**: Time course of % CBF increase (mean ± SEM) during whiskers stimulations (◼) in tPANull mice (**D**) and their

littermate (**C**), before (**—**/**—**) and after (**—**/**—**) IV infusion of Ang-II. **E**: Box plots show


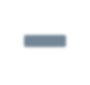

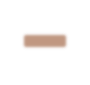

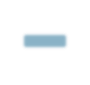

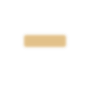


the variation of % CBF increase from baseline during whiskers stimulations in tPANull mice and their littermate before and after IV infusion of Ang-II. Box plot with medians, 1st and 3rd quartiles, min and max with values for each mouse. *p < 0.05 from tPAWT, Kruskal-Wallis and Uncorrected Dunn’s tests, n=10 tPAWT, n=7 tPANull.


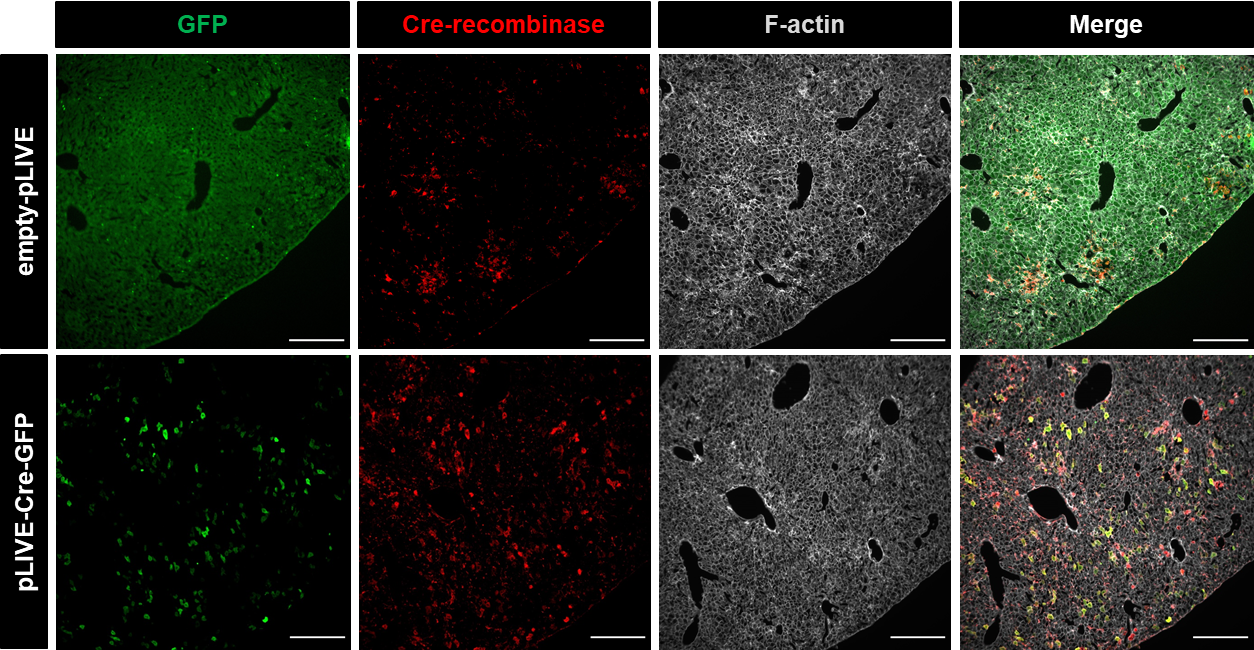

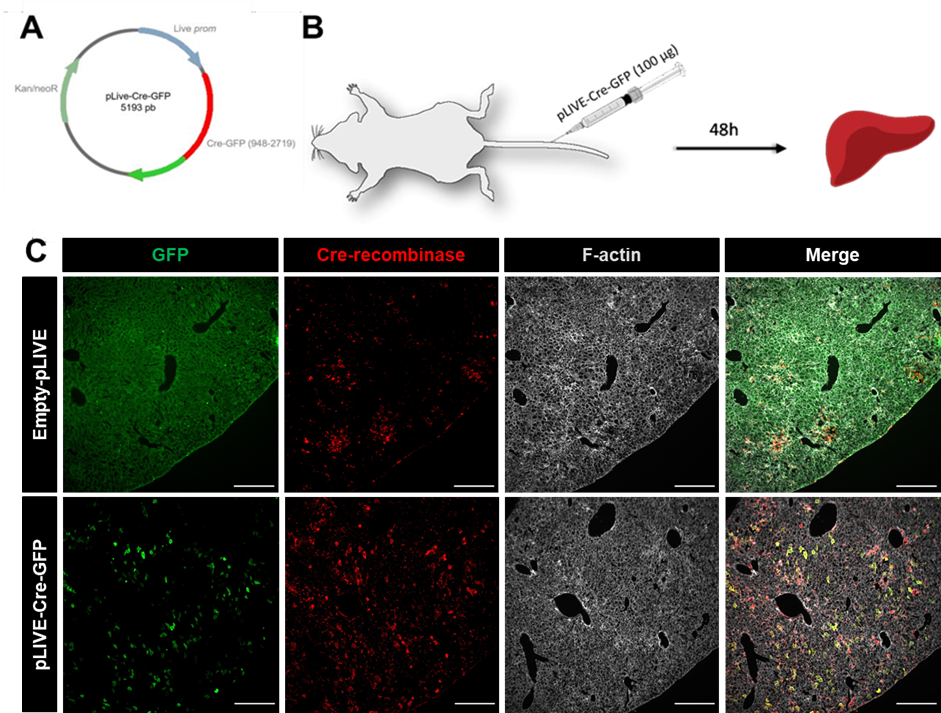

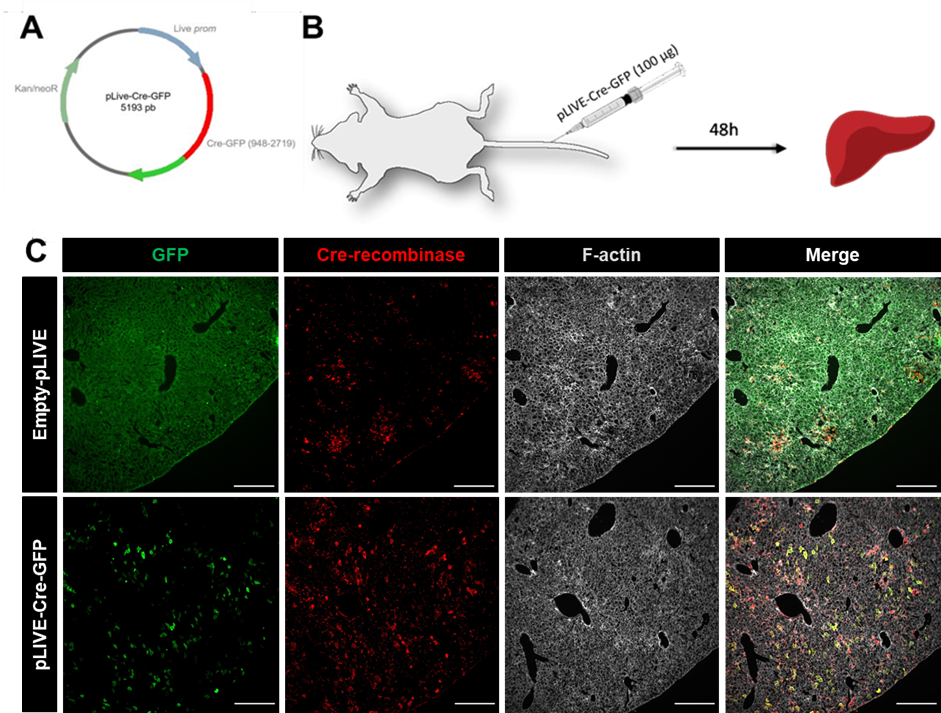


**Figure S7: Hydrodynamic transfection of pLIVE-Cre-GFP in VECad-CreΔtPA mice. A**: Construction scheme of plasmid pLIVE-Cre-GFP. The cDNA of Cre-GFP was PCR amplified from a pCAG-Cre-GFP plasmid and fused into multiple cloning sites of the pLIVE plasmid between BamHI and NotI. **B**: Experimental schematic representation of hydrodynamic transfection of empty-pLIVE or pLIVE-Cre-GFP. 48h after transfection of the plasmid, livers from transfected VECad-CreΔtPA mice were collected. **C**: Epifluorescence images of liver slices from transfected VECad-CreΔtPA mice with empty-pLIVE or pLIVE- Cre-GFP (scale bar = 500µm). Immunostaining reveals GFP (green), Cre-recombinase (red) and actin filaments with phalloidin (grey), confirming the transfection of the plasmid in hepatocytes. Images were digitally captured using a camera (CoolSNAP; Photometrics) and/or with an inverted confocal microscope (SP5, Leica). Images werevisualized respectively with Metavue 5.0 software (Molecular
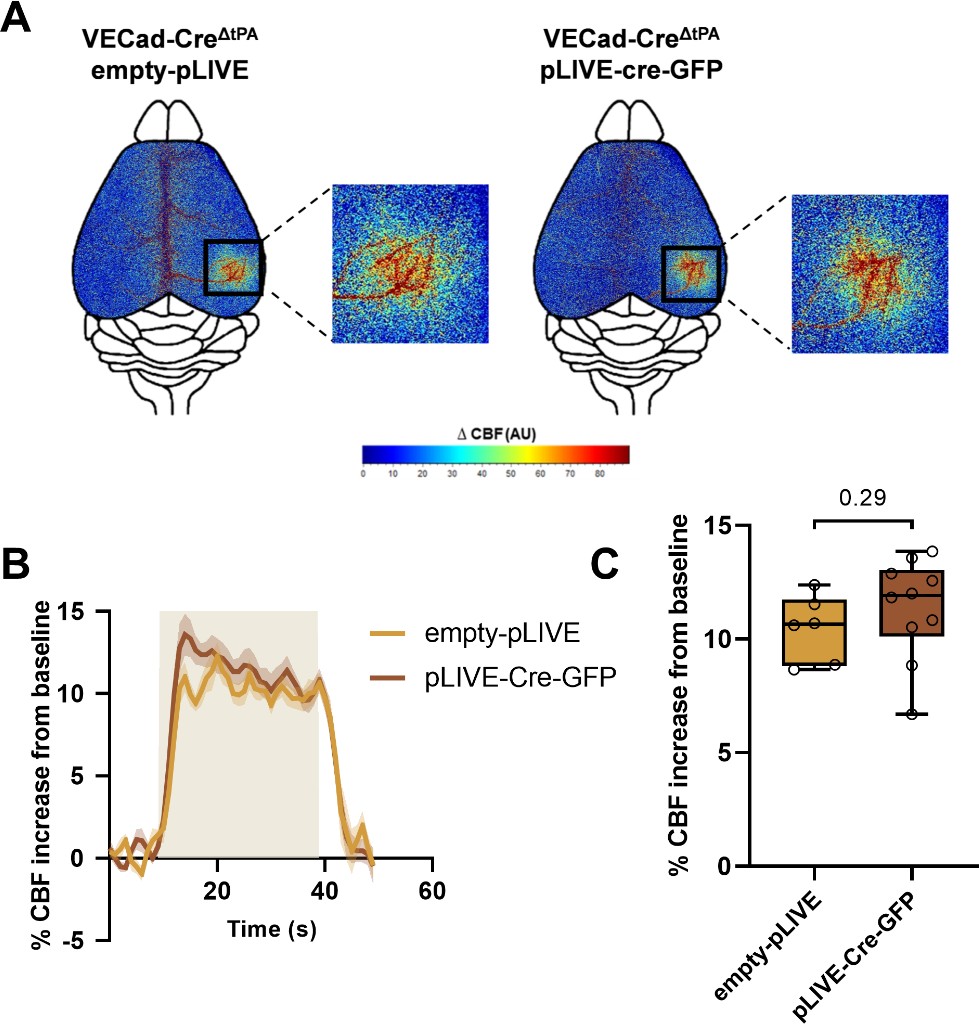
Devices, USA) and LAS AF lite software (LEICA).

**Supplementary Figure 8: Deletion of tPA in hepatocytes does not influence NVC in VECad-Cre^ΔtPA^ mice. A**: Colormap corresponds to the activation map related ∆CBF changes during whisker stimulations in VECad-CreΔtPA mice transfected with promotors empty-pLIVE or pLIVE-Cre-GFP. Warm colours indicate an elevation of CBF during whiskers stimulations. **B**: Time course of % CBF increase (mean ± SEM) during
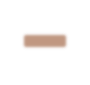

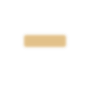
whisker stimulations (◼) of VECad-CreΔtPA transfected with empty-pLIVE (**—**) or pLIVE-Cre-GFP (**—**). **C**: Box plots show the variations of % CBF increase from baseline during whiskers stimulations of VECad-CreΔtPA mice transfected with promotors empty-pLIVE or pLIVE-Cre-GFP. Box plot with medians, 1^st^ and 3^rd^ quartiles, min and max with values for each mouse. *p < 0.05 from empty-pLIVE, Mann- Whitney test, n=6 empty-pLIVE, n=10 pLIVE-Cre-GFP.

**tPA^WT^**

**CD31**

**A**

**tPA/ CD31/ DAPI**

**tPA**

**tPA^Null^**

**Immunohistochemistry - Hippocampus**


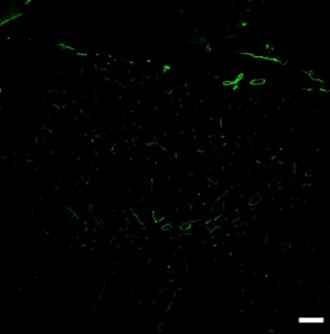

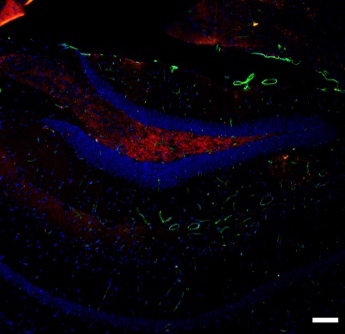

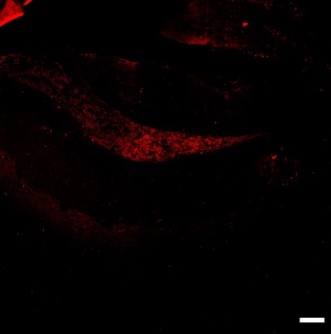

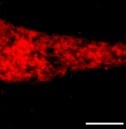

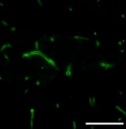

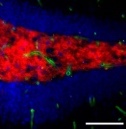


**D**

**tPA^WT^**

**CD31**

**C**

***plat/ CD31/DAPI***

***plat***

**tPA^Null^**

***In situ* Hybridization - Hippocampus**


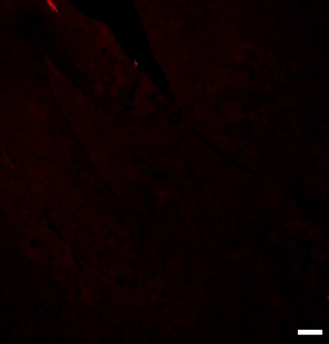

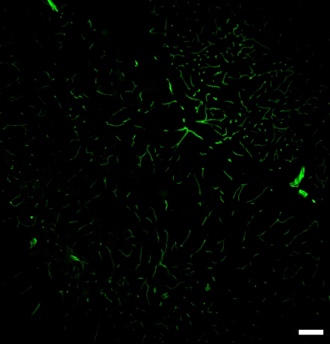

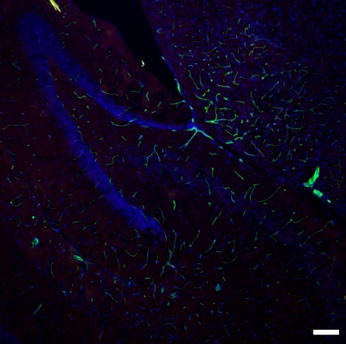

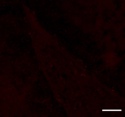

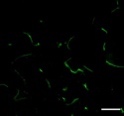

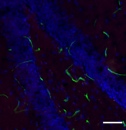

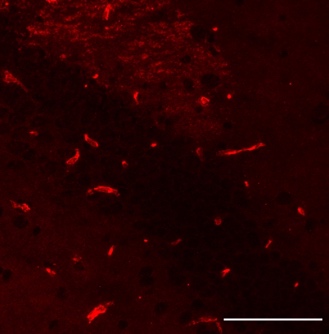

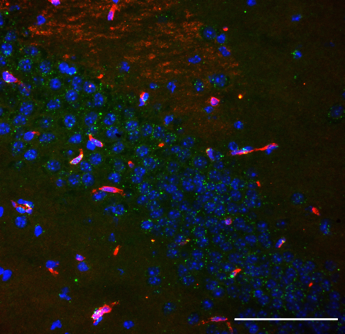

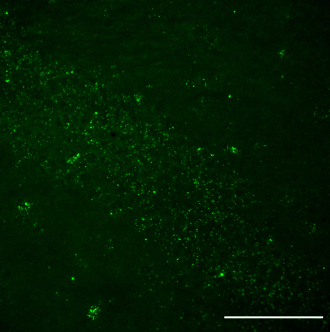

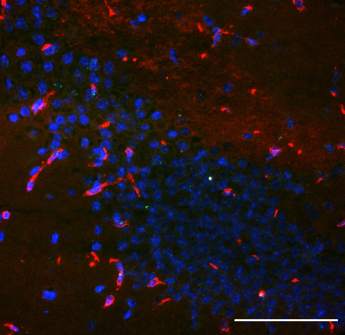

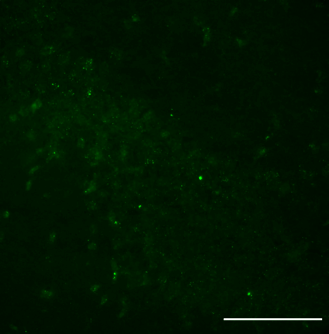

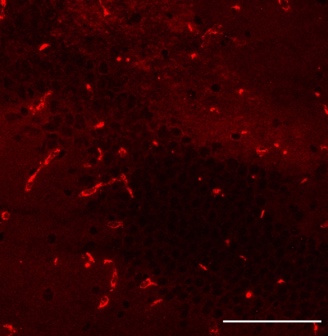

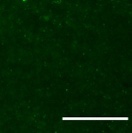

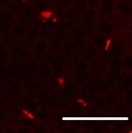

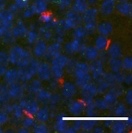

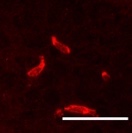

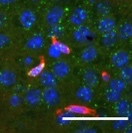

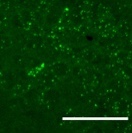


**B**

**Supplementary Figure 9: tPA expression in wild type (tPA^WT^) and tPA deficient mice (tPA^Null^). A**: Representative photomicrographs (n=5) of hippocampal tissue sections subjected to immunostainings raised against tPA (red), CD31 (green) and DAPI staining (blue); scale bar 100µm and 50µm in the magnified inserts. **B**: Quantification of tPA fluorescent intensity in hippocampus of tPA^WT^ and tPA^Null^ mice. **C**: Representative photomicrographs of hippocampal tissue sections with *plat* (gene encoding for tPA; green) revealed by *in situ* hybridization (ISH), immunostaining raised against CD31 (red) and DAPI (blue) staining; scale bar 100µm. **D**: Quantifications of *plat* positive cells in the hippocampus of tPA^WT^ (N=1; n=30) and tPA^Null^ mice (N=1; n=30).

**D**

**C**

***plat/ Neurotrace***

***plat***

***In situ* Hybridization - Cortex**

**Neurotrace**

**tPA^WT^**

**tPA^Null^**


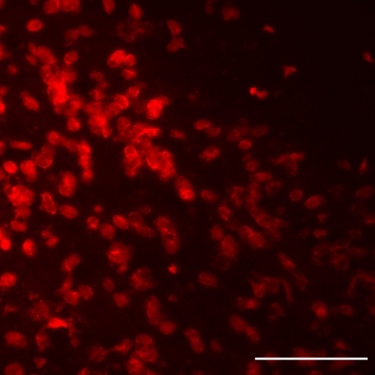

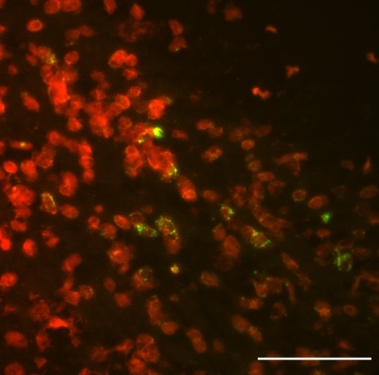

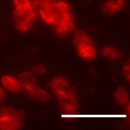

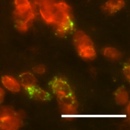

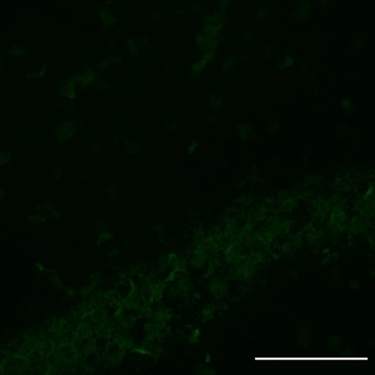

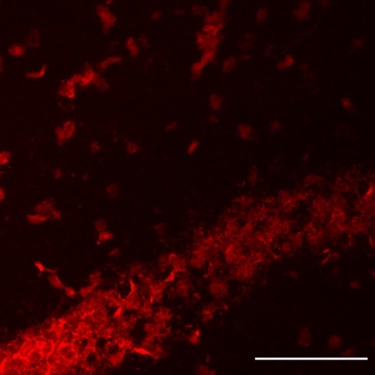

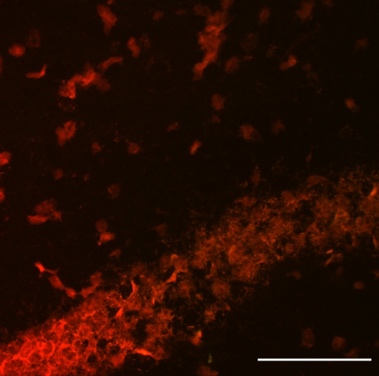

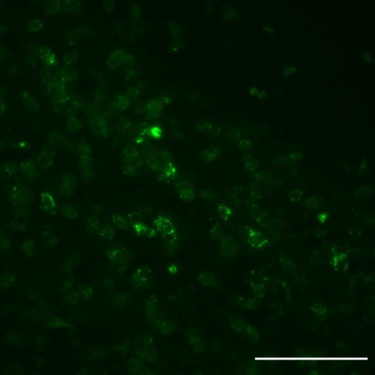

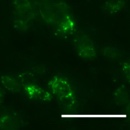


**B**

**A**

**tPA^WT^**

**tPA^Null^**

***plat/ Neurotrace***

***plat***

***In situ* Hybridization – Hippocampus**

**Neurotrace**


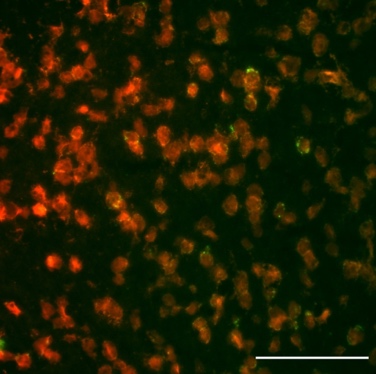

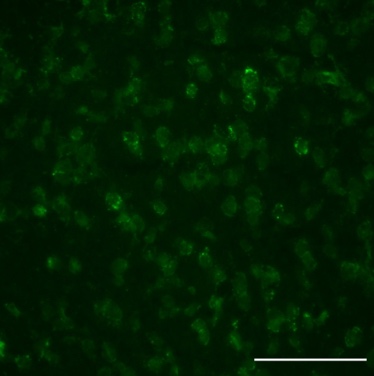

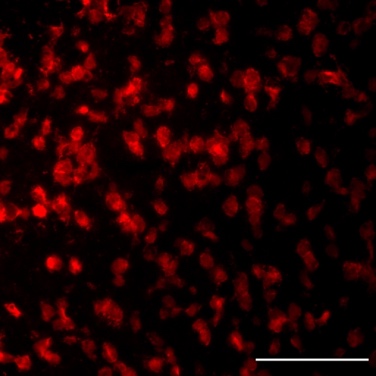

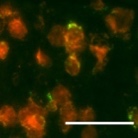

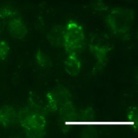

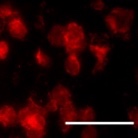

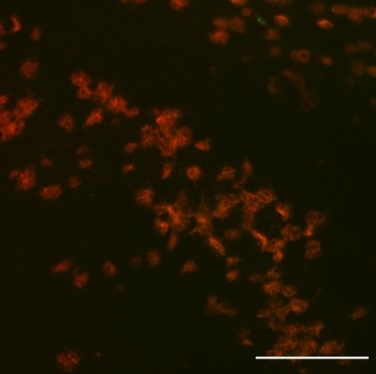

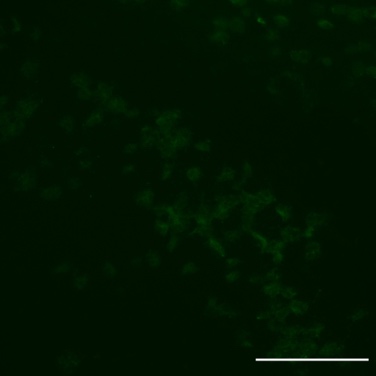

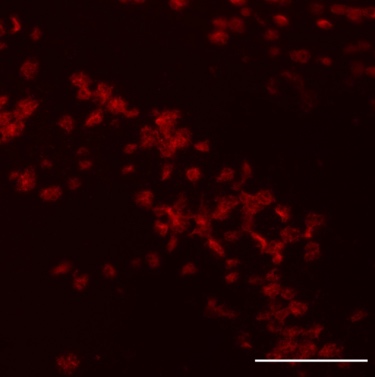

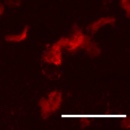

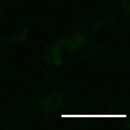

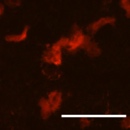

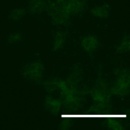

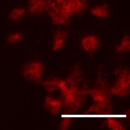

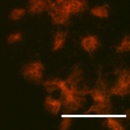


**Figure 10: tPA expression in wild type (tPA^WT^) and tPA deficient mice (tPA^Null^). A**: Representative photomicrographs of hippocampal tissue sections with *plat* (*in situ* hybridization for the mRNA encoding tPA; green) and neurons labelled using neurotrace (red) stainings; scale bar 100µm. **B**: Quantifications of *plat* positive cells in hippocampus in tPA^WT^ (N=1; n=85) and tPA^Null^ (N=1; n=40) mice. **C**: Representative photomicrographs of cortical tissue sections with *plat* (*in situ* hybridization for the mRNA encoding tPA; green) and neurons labelled using neurotrace (red) stainings; scale bar 100µm. **D**: Quantifications of *plat* positive cells in the cortex of tPA^WT^ (N=1; n=100) and tPA^Null^ (N=1; n=40) mice.

**B**

**A**

**VECad-Cre^WT^**

**CD31**

***grin1/ CD31/ DAPI***

***grin1***

**VECad-Cre^ΔGluN1^**

***In situ* Hybridization - Hippocampus**


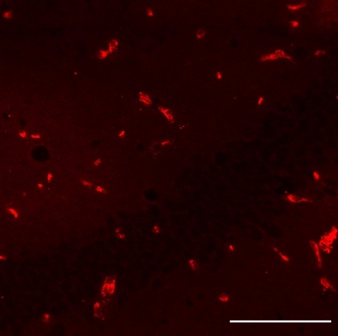

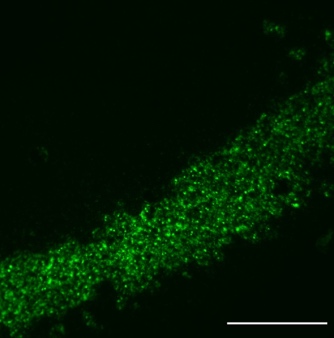

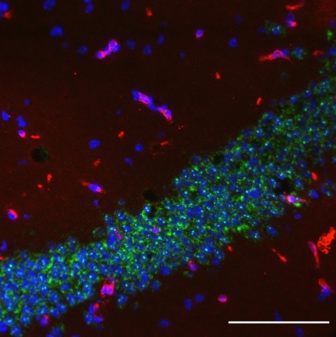

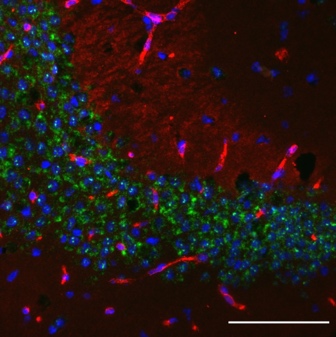

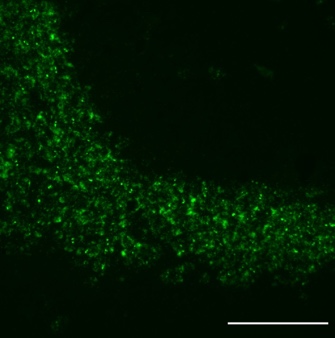

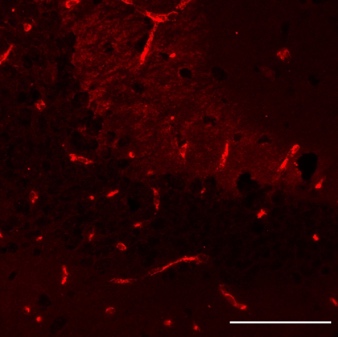

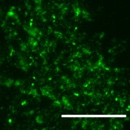

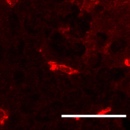

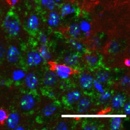

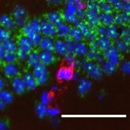

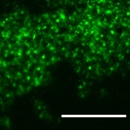

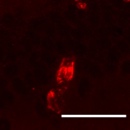


**Figure S11: Conditional deletion of GluN1 in VeCad-Cre conditional knockout mice. A**: Representative photomicrographs (n=5) of hippocampal tissue sections for *grin1* (*in situ* hybridization of the gene encoding for the GluN1 subunit of NMDA receptor; green), immunostainings against CD31 (red) and DAPI (blue) staining; scale bar 100µm. **B**: Quantifications of *grin1* positive cells in VEcad-Cre^WT^ (N=1; n=30) and VEcad-Cre ^ΔGluN1^ (N=1; n=30) mice.

**B**

**A**

**VECad-Cre^WT^**

**CD31**

***plat/ CD31/ DAPI***

***plat***

**VECad-Cre^ΔtPA^**

***In situ* Hybridization - Hippocampus**


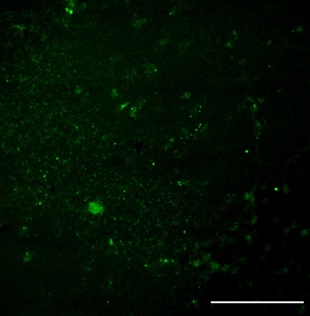

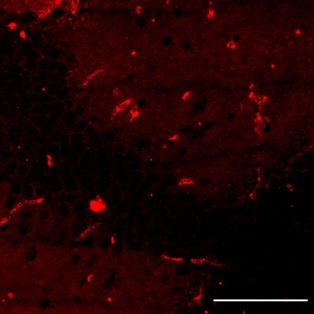

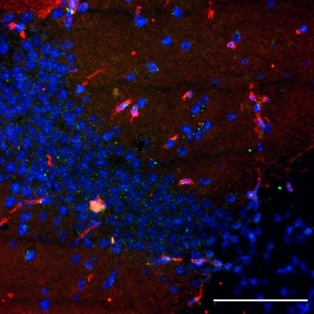

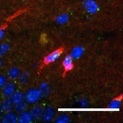

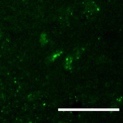

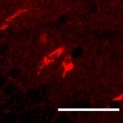

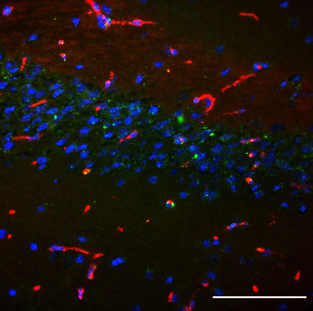

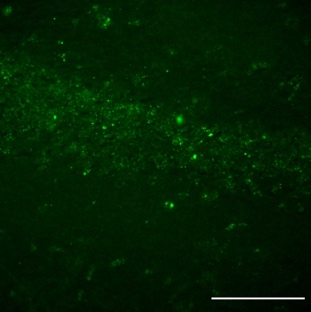

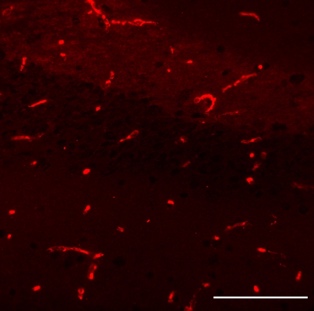

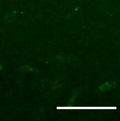

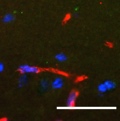

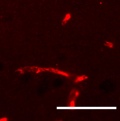


**Figure 12: Conditional deletion of tPA in VeCad-Cre conditional knockout mice. A**: Representative photomicrographs (n=5) of hippocampal tissue sections for *plat* (*in situ* hybridization of the gene encoding for tPA; green), immunostainings against CD31 (red) and DAPI (blue) staining; scale bar 100µm. **B**: Quantifications of *plat* positive cells in VEcad-Cre^WT^ (N=1; n=25) and VEcad-Cre^ΔtPA^ (N=1; n=30) mice.
